# Surface-Engineered Ru–Graphene Mesosponge Catalysts for pH-Universal and Seawater Hydrogen Evolution

**DOI:** 10.1021/acsnanoscienceau.6c00016

**Published:** 2026-03-28

**Authors:** Nichakarn Sornnoei, Naruewan Samantarkun, Thanit Saisopa, Panwad Chavalekvirat, Pawin Iamprasertkun, Bin Wang, Tawan Sooknoi, Shinichiroh Iwamura, Hirotomo Nishihara, Wisit Hirunpinyopas

**Affiliations:** † Department of Chemistry and Center of Excellence for Innovation in Chemistry, Faculty of Science, 54775 Kasetsart University, Chatuchak, Bangkok 10900, Thailand; ‡ Department of Applied Physics, Faculty of Sciences and Liberal Arts, 202946 Rajamangala University of Technology Isan, Nakhon Ratchasima 30000, Thailand; § School of Bio-Chemical Engineering and Technology, Sirindhron International Institute of Technology, and Research Unit in Sustainable Electrochemical Intelligent, 71654 Thammasat University, Pathum Thani 12120, Thailand; ∥ State Key Laboratory of Heavy Oil Processing, College of Chemistry and Chemical Engineering, China University of Petroleum (East China), Qingdao 266580, China; ⊥ Department of Chemistry, School of Science, King Mongkut’s Institute of Technology, Ladkrabang, Chalongkrung Road, Ladkrabang, Bangkok 10520, Thailand; # Faculty of Symbiotic Systems Science, and Hydrogen Energy Research Institute (HERI), Fukushima University, Fukushima 9601296, Japan; ∇ Advanced Institute for Materials Research (WPI-AIMR), 13101 Tohoku University, Sendai 9808577, Japan

**Keywords:** graphene mesosponge, ruthenium, catalyst, hydrogen, seawater

## Abstract

Efficient green hydrogen production from diverse water sources demands excellent catalysts that combine high activity, durability, and pH universality. Herein, we present a surface-engineered graphene mesosponge (GMS) uniformly decorated with ruthenium (Ru) nanoclusters as a robust electrocatalyst for the hydrogen evolution reaction (HER). The hierarchical GMS structure offers exceptional conductivity and mesoporosity, enabling nanoscale Ru dispersion and strong interfacial coupling. The obtained synergy of Ru50/GMS (optimized Ru deposition) delivers outstanding HER performance across pH range conditions, which provides overpotentials of ∼0.13 V and ∼0.061 V in acidic and alkaline electrolytes, respectively, close to those of the Pt electrode. Interestingly, Ru50/GMS achieves 10 mA cm^–2^ at only ∼0.39 V in neutral seawater, demonstrating robust operation under harsh, chloride-rich conditions. This performance is nearly 3-fold higher than that of pristine GMS, while sustaining accelerated kinetics and enhanced charge buffering. This is due to electronic modulation at the Ru–graphene interface via topological defects, which substantially optimized hydrogen adsorption and desorption, underpinning rapid reaction pathways. Furthermore, long-term operations confirm structural integrity and negligible catalyst degradation after 5000 cycles and 24 h at ultrahigh current density (−208 ± 10 mA cm^–2^), highlighting catalyst resilience for practical conditions. Therefore, this work demonstrates a scalable strategy for designing Ru-based catalysts on porous graphene supports, offering a compelling route for efficient, seawater-compatible green hydrogen production.

## Graphical abstract

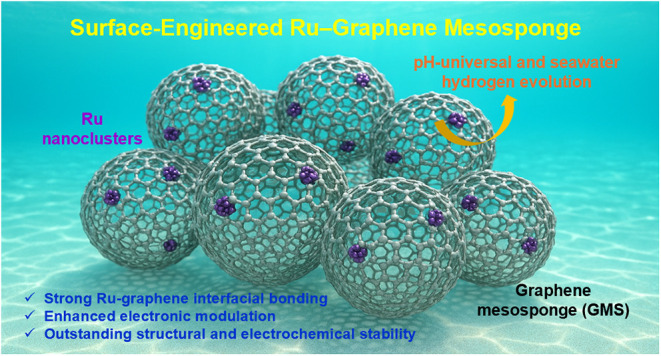

## 1 Introduction

Affordable and Clean Energy, as envisioned by Sustainable Development Goals (SDG No.7), demands transformative technologies to decarbonize global energy systems.^1^ Among these, green hydrogen produced via water electrolysis is increasingly recognized as a cornerstone for achieving net-zero emissions and sustainable fuel cycles.^1,2^ Based on the electrocatalytic hydrogen evolution reaction (HER), platinum (Pt) is defined as the benchmark catalyst due to its near optimal hydrogen binding energies, enabling balanced hydrogen adsorption and desorption kinetics.^3–5^ However, Pt-based catalysts confront critical challenges, including high cost, low abundance, and vulnerability under harsh conditions (e.g., chloride-rich environment), which affect catalyst durability and efficiency.^4–6^ These limitations hinder large-scale production and practical hydrogen generation, particularly from seawater.^4–6^ Addressing these constraints requires innovative catalyst designs that can combine high activity, resilience, and economic viability across diverse electrolytes.^2,3,7^

To overcome these challenges, alternative catalysts based on earth-abundant or less-expensive noble metals have gained considerable attention to replace Pt. Among these, ruthenium (Ru) is especially attractive due to its favorable hydrogen adsorption properties, comparatively greater availability, and significantly lower cost compared to Pt.^3,8–11^ Ru also offers unique properties for interfacial electronic modulation, which is crucial for accelerating HER activities. Moreover, recent advances have transformed from conventional nanoparticles toward atomically dispersed species and nanoclusters, leading to a powerful route to maximize atom efficiency and expose abundant active sites.^8,12–14^ The electrochemical deposition technique has proven to be a powerful strategy for achieving this transformation, giving precise control over nucleation and growth through potential-programmed cycles.^8,12–14^ This can allow systematic tuning of metal loading and dispersion on conductive material supports, optimizing interfacial coupling and electronic structure for efficient catalysts.^12–14^

Graphene-based materials have been widely considered as catalyst supports due to their good conductivity and high surface area.^9,15,16^ However, conventional two-dimensional forms produced by the chemical exfoliation (e.g., graphene oxide and reduced graphene oxide) often suffer from restacking, defect-induced instability, and limited mechanical robustness, leading to insufficient durability under prolonged operation.^16–19^ Recent studies on carbon-support Ru catalysts have highlighted their catalytic potential, which established them as highly promising candidates in advanced catalysis research.^10,20,21^ To overcome these limitations, graphene mesosponge (GMS) has recently emerged as a novel three-dimensional architecture, exhibiting hierarchical mesoporosity, seamless connectivity, and outstanding electrical and mechanical properties.^22–26^ This unique structure can facilitate uniform dispersion of catalytic species, enhancing rapid ion transport and efficient gas release, while maintaining structural integrity under harsh conditions.^25,27,28^ Consequently, GMS can serve as an excellent scaffold for anchoring noble metal nanoclusters, where its open porosity and conductivity framework enhance interfacial coupling and electronic modulation.^27,28^ These attributes could be potentially advantageous for achieving HER activity across pH-universal and chloride-rich conditions, positioning GMS as a next-generation catalyst support.

In this work, we present a surface-engineered GMS uniformly anchored with Ru nanoclusters (Ru/GMS) as a robust HER catalyst for pH-universal and seawater electrolysis. The obtained GMS was synthesized via a template-assisted chemical-vapor deposition and subsequently graphitized at high temperature to achieve a mechanical property, a hierarchically porous network. The GMS was obtained with a multilayer structure (2–3 layers), which possesses high electrical conductivity and superior oxidation resistance, leading to excellent structural integrity when compared to single-layer graphene frameworks. Ru nanoclusters were introduced using a controlled electrochemical deposition technique, enabling precise tuning of Ru loading and homogeneous dispersion across the GMS surface. Comprehensive structural and electronic characterizations, e.g., transmission electron microscopy (TEM), scanning transmission electron microscopy-energy dispersive X-ray spectroscopy (STEM-EDS) mapping, X-ray diffraction (XRD), Raman spectroscopy, X-ray photoelectron spectroscopy (XPS), and surface analysis, confirm nanoscale Ru distribution, preserved GMS crystallinity, and strong Ru–graphene interfacial coupling. Electrochemical evaluations reveal that Ru/GMS exhibits outstanding HER performance across acidic, alkaline, and neutral seawater electrolytes, achieving low overpotentials and excellent HER kinetics (low Tafel slopes). The structural integrity of Ru/GMS was maintained under prolonged cycling and ultrahigh current density, indicating superior long-term catalyst stability. This work can establish a scalable catalyst design that integrates porous graphene frameworks with tailored Ru nanoclusters, offering a compelling route toward efficient and durable seawater green hydrogen production.

## 2 Experimental Section

### 2.1 Materials

Graphene mesosponge (GMS, 2–3 layers) was supplied by 3DC Inc. Isopropanol and Nafion perfluorinated resin solution (5 wt %) were purchased from Sigma-Aldrich. Ruthenium­(III) chloride hydrate (99.99%, RuCl_3_·*x*H_2_O, Premion) was purchased from Thermo Fisher Scientific. A graphite rod electrode (6/70 mm) was purchased from Redox.me. Glassy carbon disk (3 mm diameter) and silver/silver chloride reference electrodes were purchased from PalmSens. Ultrapure deionized water (Milli Q water) was used in all aqueous solution preparation.

### 2.2 Synthesis of Graphene Mesosponge

Graphene mesosponge (GMS, 2–3 layers, 3DC Inc.) was synthesized via chemical-vapor deposition (CVD) process employing porous MgO nanoplatelets as a template and CH_4_ as the carbon precursor, as shown in Figure 1a.^22,23^ The resulting carbon-coated MgO was then treated with 5 M HCl to remove the MgO template, resulting in a carbon mesosponge (CMS). Finally, CMS was graphitized under vacuum at 1800 °C for 1 h, producing GMS with a highly interconnected porous network (see Figure 1b).

**Figure 1:**
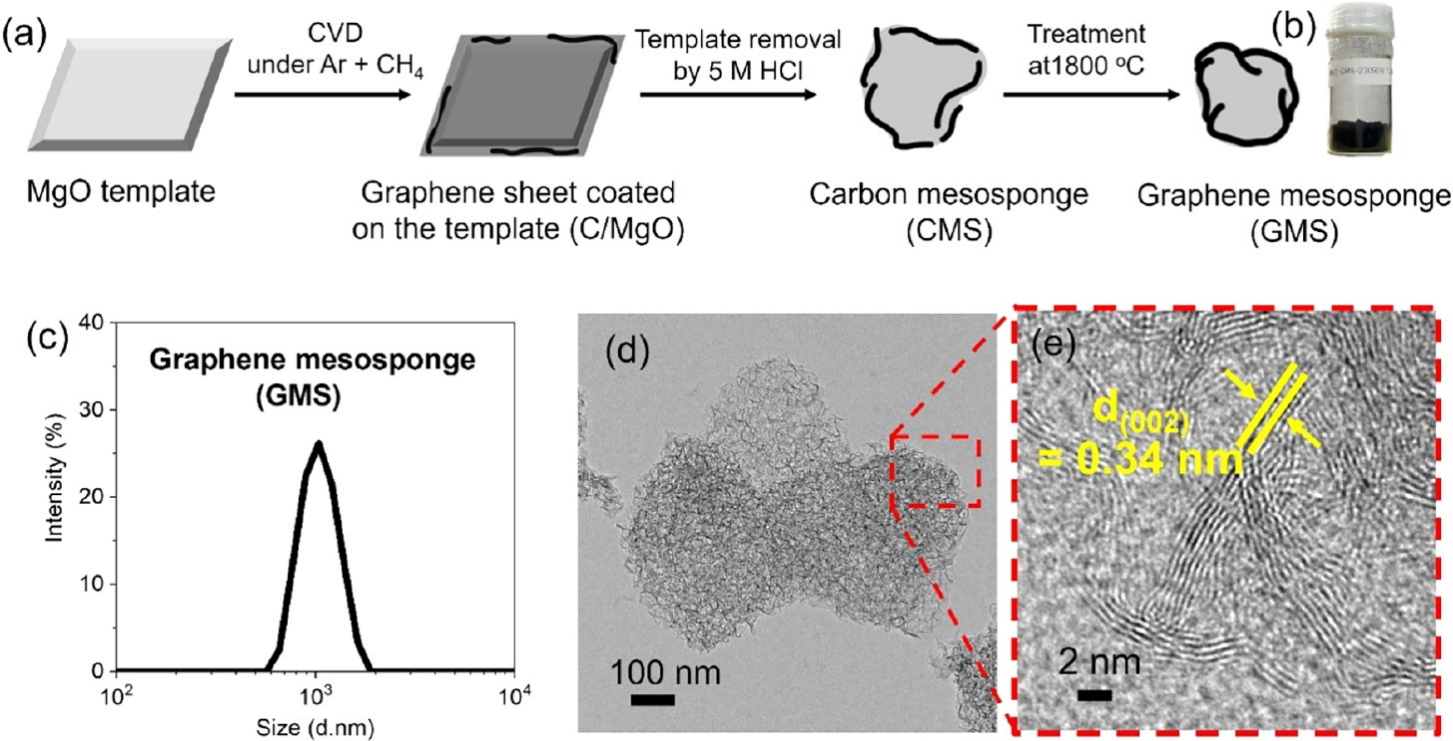
Synthesis of graphene mesosponge (GMS). (a) Schematic illustration of the chemical-vapor deposition (CVD) using MgO as a template. (b) Photographs of the obtained GMS powder. (c) Particle size distribution of the GMS, confirming homogeneous dimensions. (d) TEM image of GMS with (e) HR-TEM image showing the characteristic (002) diffraction of graphene.

### 2.3 Electrodeposition of Ru Nanoclusters

To prepare the graphene mesosponge (GMS) ink, 5 mg of GMS was dispersed in 950 μL of a mixture of 2-propanol and water (1:1 v/v) with 50 μL of Nafion solution, giving a concentration of 5 mg mL^–1^. For electrode preparation, 1.42 μL of the GMS ink was dropped onto a polished glassy carbon electrode (GCE) and dried at 55 °C, used as a working electrode for subsequent Ru deposition.

Electrodeposition of Ru nanoclusters was performed with a three-electrode configuration using a potentiostat (PalmSens4, PalmSens BV, Netherlands) under ambient conditions. The GMS on a GCE, a graphite rod, and Ag/AgCl (3 M KCl) electrodes were used as the working, counter, and reference electrodes, respectively. Briefly, 1 mM RuCl_3_ as an electrolyte was prepared by dissolving RuCl_3_·*x*H_2_O in a 0.1 M H_2_SO_4_ solution. The electrolyte was purged with nitrogen for 30 min prior to the electrochemical process. The Ru deposition was achieved via linear sweep voltammetry (LSV) from a potential of −0.5 to 0.4 V vs Ag/AgCl at a scan rate of 20 mV s^–1^.^8^ The amount of Ru content on GMS support was controlled by varying the number of LSV cycles at 2 cycles, 10 cycles, 20 cycles, 50 cycles, 100 cycles, and 150 cycles, at which the samples were denoted as Ru2, Ru10, Ru20, Ru50, Ru100, and Ru150, respectively (Figure S1). Then, the electrodes were rinsed thoroughly with ultrapure water and dried at 55 °C for 30 min before the catalytic evaluation.

### 2.4 Characterization of Graphene Mesosponge

#### 2.4.1 Morphology and Physical Properties

The particle size distribution of the GMS was determined using dynamic light scattering (DLS, Malvern Panalytical Zetasizer Advance) operating with a 633 nm He–Ne laser. ζ-Potential measurements were performed by using a disposable folded capillary cell. Morphological and structural properties of GMS were examined by transmission electron microscopy (TEM; JEOL JEM-2010F) operated at an accelerating voltage of 200 kV. For TEM specimen preparation, the GMS was initially deposited on conductive Toray carbon paper for Ru electrodeposition, then redispersed in an isopropanol and water mixture prior to being dropped onto a copper grid, and dried in a vacuum oven.

X-ray diffraction (XRD; Bruker D8 ADVANCE diffractometer) with Cu Kα radiation (λ = 1.5406 Å) was used to determine crystalline phases. The diffraction patterns were recorded over a 2θ range of 5–70° with a step size of 0.02° s^–1^. A scanning electron microscope (SEM; FEI Quanta 450 FEG) at an accelerating voltage of 15 kV was used to determine the surface morphology of Ru/GMS catalysts. X-ray photoelectron spectroscopy (XPS; Kratos AXIS Supra) was used to analyze the electronic structure and chemical states of Ru/GMS samples at varied Ru loading. The surface properties, including surface area and porosity, of Ru/GMS samples were determined using nitrogen adsorption–desorption isotherms (Micromeritics 3Flex). Raman spectroscopy was used to determine the structure and bonding characteristics of Ru/GMS (HORIBA XploRA PLUS). Raman spectra were recorded in the range between 500 and 3500 cm^–1^ using a 532 nm laser (2.33 eV) with a grating of 1200 l/mm (750 nm) with a 100× objective lens.

#### 2.4.2 Electrochemical Measurement

Hydrogen evolution reaction (HER) performance was evaluated in a three-electrode configuration under acidic (0.5 M H_2_SO_4_), alkaline (1 M KOH), and neutral seawater electrolytes. All electrolytes were purged with nitrogen for 30 min prior to measurements. Catalytic activities were determined using LSV at a scan rate of 5 mV s^–1^. The overpotential at 10 mA cm^–2^ and Tafel values were used to evaluate the catalytic activity. Electrochemical impedance spectroscopy (EIS) was conducted at −0.13 V vs RHE at an amplitude of 10 mV over a frequency range of 10 mHz to 0.1 MHz to determine the solution resistance (*R*_s_) and charge transfer resistance (*R*_ct_). All potentials were converted to the reversible hydrogen electrode (RHE; *E*_(RHE)_ = *E*_(Ag/AgCl)_ + 0.207 + 0.0591pH). All polarization curves were corrected with 90% *iR* compensation.

Electrochemical double-layer capacitances (*C*_dl_) were estimated using cyclic voltammetry (CV) in nonfaradaic regions at varied scan rates (5–200 mV s^–1^). The durability of the catalysts was evaluated with LSV before and after 5000 CV cycles within the potential range of 0.7–0.9 V at 100 mV s^–1^ in acidic media. Long-term stability was further examined under high current density using a chronoamperometry at −0.3 V vs RHE for 24 h in acidic media.

## 3 Results and Discussion

### 3.1 Morphology and Structure of GMS

As shown in Figure 1c, the particle size distribution of graphene mesosponge (GMS) determined by dynamic light scattering was observed to be *ca*. 500–2000 nm, indicating a well-defined mesoscale architecture. The ζ-potential of GMS was measured to be about −15 mV, suggesting a moderately negative surface charge that can facilitate the adsorption and subsequent electrodeposition of Ru species. Figure 1d shows the TEM image of pristine GMS, revealing a distinctive sponge-like morphology with a highly porous framework, which results from interconnected graphene sheets. The framework consists of thin and wrinkled layers forming hierarchical mesopores, which are advantageous for rapid ion diffusion and efficient mass transport in various electrochemical applications.^24^ Such a unique architecture provides a large accessible surface area for the decoration of the catalytic species. High-resolution TEM image further confirms the graphitic nature of the carbon materials, as shown in Figure 1e. It is clearly seen that GMS possesses well-defined lattice fringes with an interlayer spacing of ∼0.34 nm, corresponding to the (002) plane of turbostratic carbon. The high degree of graphitization obtained in GMS is essential for achieving superior electrical conductivity, mechanical robustness, and chemical stability, which are advantageous for catalyst–support materials in electrochemical applications, particularly under harsh operating conditions.

### 3.2 Morphology and Structure of Ru/GMS

Figure 2 shows comprehensive morphological and elemental analyses of the Ru-decorated graphene mesosponge with 50 cycles for Ru depositions (denoted as Ru50). The morphology of Ru50 is shown by TEM images in Figure 2a,b. It was found that GMS support still retains its unique sponge-like structure with a highly interconnected graphene network, demonstrating structural robustness even after deposition of Ru nanoclusters. This is confirmed by the graphitic nature of the GMS, exhibiting distinct lattice fringes with an interlayer spacing of ∼0.34 nm, which corresponds to the (002) plane of turbostratic carbon (Figure 2c). Figure 2d–g shows the backscattered electron TEM and elemental mapping images of Ru50, indicating a homogeneous distribution of carbon, oxygen, and ruthenium across the GMS framework. The presence of oxygen likely originates from surface oxidation formed during Ru nucleation (discussed later in Raman and XPS analysis). Notably, we found that Ru signals appear relatively sparse, suggesting nanoscale dispersion rather than aggregation. This could be advantageous for maximizing catalytic active sites for efficient hydrogen evolution reaction (HER). Comparative elemental mappings for Ru2, Ru50, and Ru150 samples are shown in Figure S2, further demonstrating progressive Ru enrichment with increasing Ru loading. Moreover, the scanning transmission electron microscopy (STEM) images in Figure 2h,i clearly demonstrate the presence of Ru nanoclusters uniformly anchored on the graphene surface with a median size of ∼8 nm (Figure 2j). This observation is further supported by the high-resolution STEM image in Figure 2i, which reveals well-defined lattice fringes with an interlayer spacing of ∼0.21 nm, corresponding to the (101) plane of metallic Ru. These results can confirm the crystalline nature of the Ru nanoclusters embedded within the conductive GMS framework.

**Figure 2:**
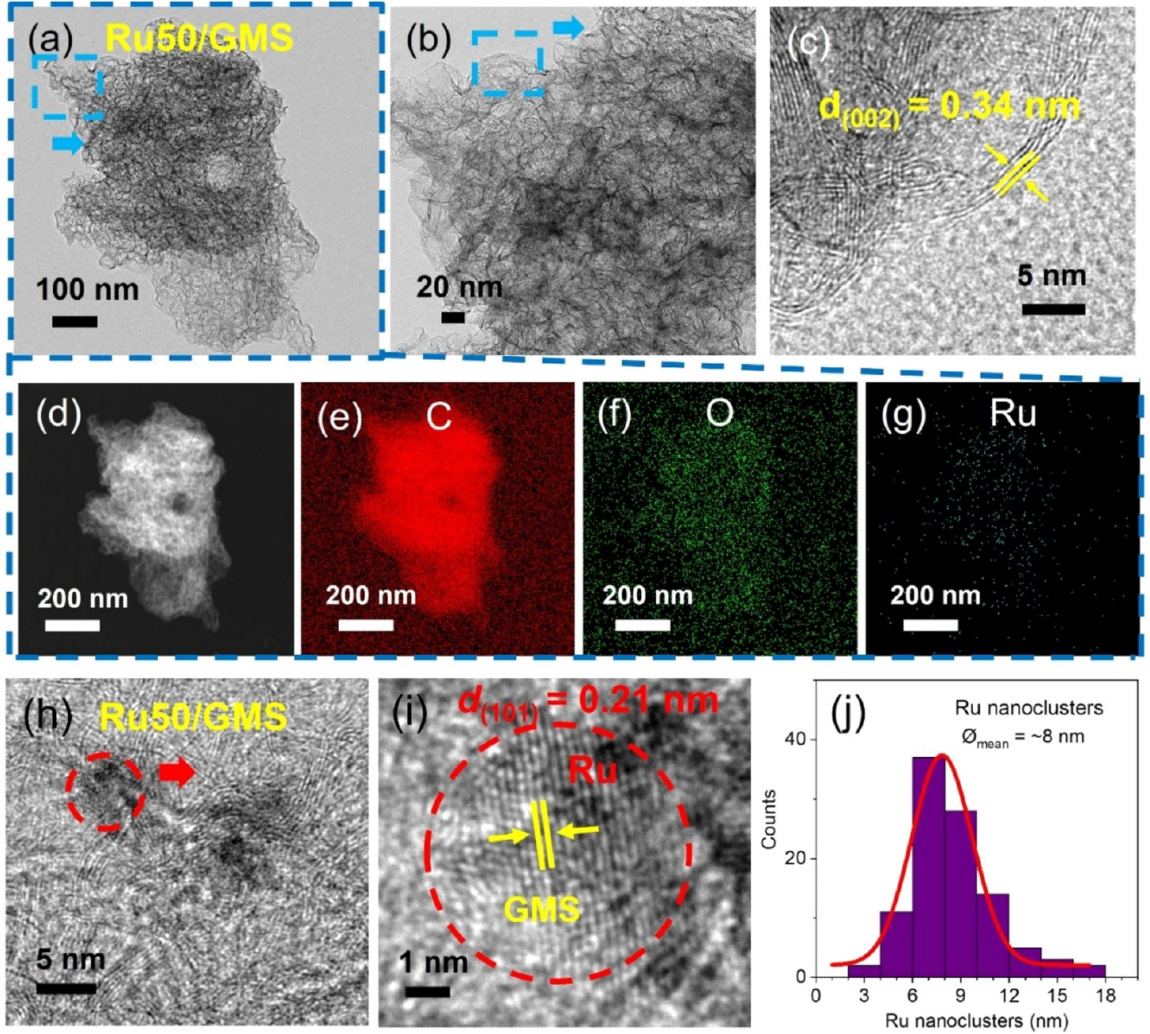
(a) TEM images of Ru50/GMS (Ru50) and (b, c) enlarged TEM images showing the characteristic (002) lattice fringes of graphene. (d) Backscattered electron TEM image of Ru50 with corresponding elemental mapping showing (e) carbon, (f) oxygen, and (g) ruthenium. (h) STEM image of Ru50 and (i) Ru nanocluster anchored on the GMS surface with a lattice spacing of ∼0.21 nm, corresponding to the (101) plane of metallic Ru. (j) Size histogram of Ru nanoclusters as measured by statistical TEM data, showing a median size of Ru nanoclusters of ∼8 nm.

Figure 3 provides morphological insights into pristine GMS and Ru-modified GMS (Ru/GMS) electrodes. In Figure 3a, the SEM image of the pristine GMS reveals a highly porous sponge-like architecture. The interconnected graphene framework can be clearly observed in the enlarged view, as shown in Figure 3b. This hierarchical structure offers an extensive surface area and abundant porosity, which can potentially serve as a promising scaffold for anchoring Ru nanoclusters and facilitating efficient mass transfer during electrochemical activities.^22,23,25^ To quantify Ru incorporation, energy-dispersive X-ray spectroscopy (EDX) was employed on Ru/GMS samples at varying Ru loading. The Ru content as a function of deposition cycles is shown in Figure 3c. The Ru contents were determined to be 0.14 wt % (Ru2), 7.9 wt % (Ru10), 15.7 wt % (Ru50), 19.1 wt % (Ru100), and 27.8 wt % (Ru150). It was clearly seen that the trend exhibits a nearly linear increase in Ru content with cycle number (*R* ^2^ = 0.89), indicating that additional electrodeposition cycles progressively enrich the Ru loading, particularly on the outer electrode surface. This precise controllability enables systematic tuning of the metallic Ru density on the GMS scaffold, thereby optimizing the availability of catalytic active sites for enhanced HER performance. The presence of Ru species was also observed by X-ray fluorescence spectroscopy, exhibiting the increase in Ru intensity at higher Ru deposition (see Figure S3). To obtain a bulk quantification, the total Ru content of the catalyst was determined by inductively coupled plasma-optical emission spectroscopy (ICP-OES), which provided a Ru loading of 1.9 wt % for Ru50. It should be noted that ICP-OES reflects the Ru content of the entire electrode (inner and outer electrode material), whereas EDX preferentially probes the outer electrode surface (Figure 3c). These complementary methods provide both the relative surface trend and total Ru content.

**Figure 3:**
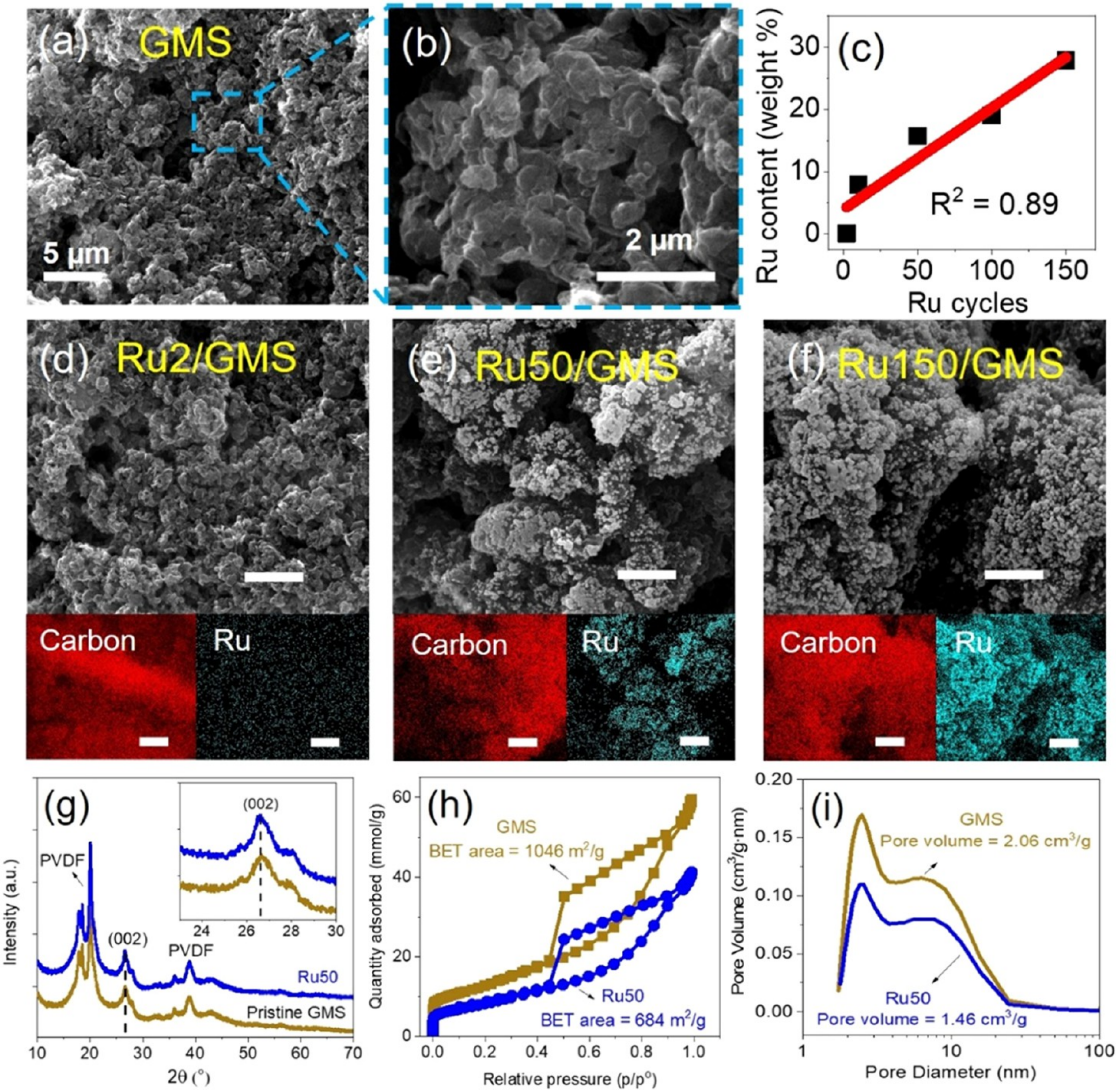
Morphological and structural characterizations. SEM images of (a) pristine GMS with (b) its enlarged image. (c) Ru content as a function of electrodeposition cycles showing a good correlation with *R* ^2^ of 0.89. SEM images and elemental mapping of (d) Ru2, (e) Ru50, and (f) Ru150, showing the nanocluster formation on the GMS scaffold. All scale bars are 5 μm. (g) Wide-angle PXRD pattern of Ru50 and pristine GMS, with inset highlighting the (002) diffraction peak of graphene. Note: XRD samples were prepared on PVDF filters. (h) Nitrogen adsorption–desorption isotherms of Ru50 and pristine GMS and (i) their corresponding pore-size distributions as determined by the BJH adsorption method.

To elucidate morphological changes upon Ru deposition, SEM images and corresponding elemental mapping for Ru2, Ru50, and Ru150 are shown in Figure 3d–f. At low Ru loading (Ru2), the GMS surface still remains largely exposed, resembling the pristine structure (Figure 3d). Elemental mapping displays a sparse Ru distribution on GMS support, indicative of the formation of isolated nanoclusters without significant aggregation. At optimized Ru loading (Ru50), the SEM image shows partial surface coverage by Ru nanoclusters, observed as brighter regions in Figure 3e. This is corroborated by elemental mapping, which demonstrates well-uniformed Ru dispersion across the GMS network. Additional SEM images and elemental mappings for Ru10 and Ru100 are provided in Figure S4. In contrast, at high loading (Ru150), the SEM image shows a large amount of Ru clusters on the GMS surface, indicating dense Ru coverage (Figure 3f). This can be clearly seen by elemental mapping, which confirms substantial Ru enrichment. Although increased Ru depositions can distinctly enhance catalytic site density, excessive coverage may partially obstruct mesopores, resulting in limitations in mass transport and ion diffusion during HER kinetics. Our findings underscore the importance of optimizing Ru loading to balance active site density and structural accessibility, thereby achieving superior catalytic performance (discussed later).

Figure 3g shows the wide-angle powder X-ray diffraction (PXRD) patterns of pristine GMS and Ru50. It was observed that both samples exhibit a prominent diffraction peak at ∼26.6°, corresponding to the (002) plane of turbostratic carbon. This suggests that the graphene-stacking structure is retained even after Ru deposition (Figure 3g, inset). It can be implied that Ru nanoclusters were anchored on the GMS surface without intercalating into the graphene layers, as evidenced by the absence of any shift in the (002) diffraction pattern. Importantly, we have not found distinct Ru-related peaks (JCPDS 06–0663: hexagonal Ru structure), indicating that Ru exists predominantly on the GMS surface as highly dispersed nanoclusters rather than forming large crystalline aggregates. This agrees well with a previous work studying low-loaded Ru nanoparticles on a titanium oxynitride-graphene oxide.^21^ This finding is consistent with the TEM observations, as discussed in Figure 2 (median size of ∼8 nm for Ru nanoclusters). It should be noted that the XRD samples were prepared on PVDF filters, which were also used as a referencing peak at 2θ of 20.17°.^19^

To evaluate changes in surface area and porosity after Ru deposition, Figure 3h shows the nitrogen adsorption–desorption isotherms of pristine GMS and Ru50. It was clearly seen that both samples exhibit pronounced hysteresis loops classified as type-IV isotherms, which demonstrate characteristics of mesoporous structures. According to Brunauer–Emmett–Teller (BET) analysis, the specific surface area of pristine GMS and Ru50 was determined to be *ca*. 1046 and 684 m^2^ g^–1^, respectively. It was found that the BET surface area of Ru50 markedly decreased, accompanied by a reduction in the total pore volume from 2.06 cm^3^ g^–1^ for pristine GMS to 1.46 cm^3^ g^–1^ for Ru50. The cumulative pore volumes of GMS and Ru50, determined using the BJH method from the adsorption branch of the isotherms, further confirm these changes (Figure 3i). The observed decreases in surface area and pore volume are primarily attributed to partial pore blockage arising from the deposition of Ru nanoclusters, which occupy portions of the internal pore networks. It should be noted that although a fraction of the mesopores is partially occupied by Ru species, the overall hierarchical pore structure of Ru50 remains largely preserved, as evidenced by the retained mesoporous distribution (Figure 3i). These results clearly demonstrate that the mesoporous backbone (2–30 nm range) is maintained, which is crucial for facilitating rapid electrolyte diffusion and efficient gas release during HER kinetics.

Figure 4a shows the Raman spectra of the pristine GMS and Ru/GMS samples. It was found that all spectra exhibit three dominant peaks at ∼1330 cm^–1^, ∼1570 cm^–1^, and ∼2660 cm^–1^, corresponding to the *D*, *G*, and 2*D* bands, respectively.^22,23,27^ The *G* band arises from the in-plane stretching of the sp^2^-hybridized carbon systems, while the *D* band originates from edge sites, lattice disorder, structural imperfections, or topological defects.^15,29^ It should be noted that GMS materials are characterized by an extremely low density of edge sites; consequently, the observed *D* band intensity is predominantly attributed to topological defects (i.e., five and seven-membered carbon rings) rather than edge-related disorder.^30^ The intensity ratio of the *D* band to *G* band (*I*_D_/*I*_G_) represents a key indicator for assessing structural disorder and defect density.^15,29^ A relatively high *I*_D_/*I*_G_ ratio of pristine GMS (∼1.50) indicates abundant topological defects,^22,23,27^ which are advantageous as anchoring sites for catalytic metal deposition. This suggests that the GMS framework is totally different from conventional edge-defect-rich graphene or reduced graphene oxide (rGO), which are highly reactive but less oxidation-resistant, often leading to corrosion, particle detachment, and agglomeration under catalytic activities.^23,27,30^ Indeed, oxygen content of GMS (0.58% as determined by XPS analysis) is substantially lower than that typically observed in GO/rGO families (>40%), preserving superior electrical conductivity and stability in aqueous solutions.^15,19,31^ Upon Ru incorporation, the *I*_D_/*I*_G_ ratio increases slightly to ∼1.56, suggesting that Ru species are predominantly anchored at topological defects. Additionally, the *D* band enhancement can arise from surface-enhanced Raman scattering, which is attributed to localized electromagnetic field amplification and charge transfer contribution at interfacial Ru-GMS junctions.^32^ Topological defects, such as edge sites, can serve as effective anchoring sites for the deposition of metal nanoparticles, while they exhibit substantially higher resistance to electrochemical oxidation compared to edge sites. This distinction positions GMS as a highly durable catalyst support.^28,30^ Whereas single-graphene GMS typically exhibits an intense 2D band that is comparable to or even stronger than the *G* band, the GMS used in this study shows a much weaker 2D band.^22,26^ This observation further supports the formation of highly crystalline graphene walls consisting of 2–3 layers, in good agreement with TEM analysis in Figure 2. Such a multilayer GMS structure is advantageous for achieving enhanced electrochemical stability.

**Figure 4:**
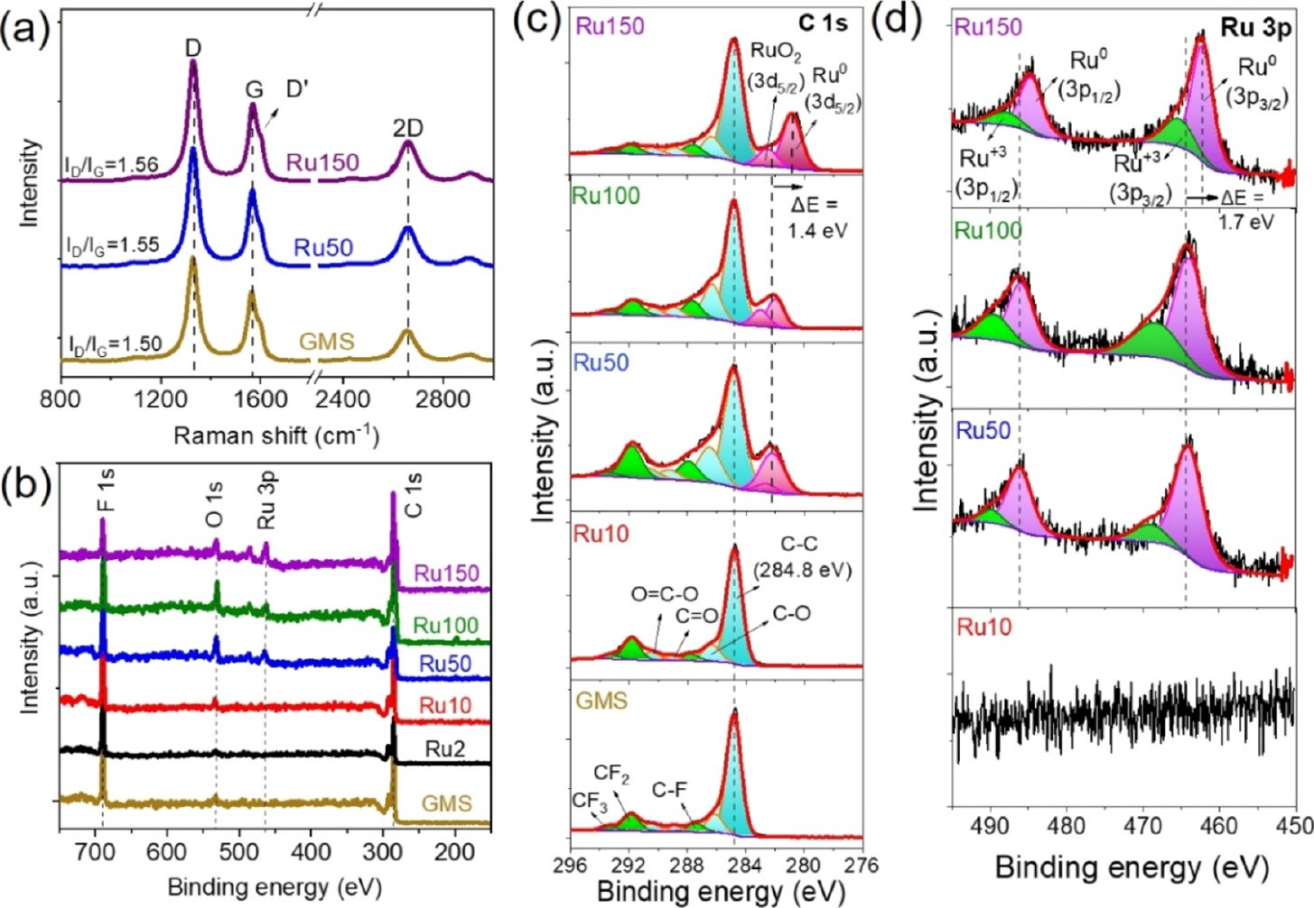
Physical properties of Ru/GMS. (a) Raman spectra of Ru/GMS (Ru50 and Ru150) and pristine GMS. (b) XPS survey spectra of pristine GMS and Ru/GMS at varied Ru loadings (Ru2–Ru150). High-resolution XPS spectra of (c) C 1s and (d) Ru 3p regions for Ru/GMS samples and pristine GMS. All XPS spectra were calibrated using the C 1s peak of advantageous carbon at 284.8 eV. Note samples were prepared on a conductive carbon substrate (Teflon-treated carbon fiber paper) for sample characterizations.

To further confirm Ru incorporation and analyze surface chemistry, X-ray photoelectron spectroscopy (XPS) was performed on Ru/GMS samples at varied Ru loadings. All XPS spectra were calibrated with C 1s at 284.8 eV, and peak deconvolution was conducted with CasaXPS software. Figure 4b shows the XPS survey spectra of the pristine GMS and Ru/GMS samples (Ru2–Ru150). All spectra exhibit characteristic signals for C 1s (284.8 eV), O 1s (531.8 eV), and Ru 3p (∼463–485 eV), confirming a successful incorporation of Ru species into the GMS network. It was found that the intensity of Ru 3p peaks progressively increases with higher Ru deposition cycles (particularly evident for Ru50), indicating systematic enrichment of Ru content. This agrees well with the EDX analysis and elemental mapping results, as discussed in Figure 3c–f. Note that a distinct F 1s peak at ∼690 eV originates from the Teflon-treated carbon fiber used as the substrate for preparing XPS samples. Additionally, XPS analysis reveals a pronounced evolution of surface oxygen content upon Ru deposition. We found that the oxygen content increases markedly from 0.58 at% in pristine GMS to 1.96 at% for Ru10 and reached a maximum of 8.65 at% for Ru50, before decreasing to 5.02 and 3.63% at% for Ru100 and Ru150, respectively (see Figure S5). The nonlinear trend indicates that the observed oxygen species are mainly associated with the dispersion and surface chemistry of Ru nanoclusters rather than originating from oxidation of the GMS framework itself. It should be noted that the exceptional oxidation resistance of GMS has been previously reported in energy storage and electrocatalytic studies.^26–28,30^ This stability is attributed to its highly graphitized 3D graphene framework, which effectively suppresses oxidation degradation under electrochemical conditions. The substantial enrichment of oxygen at low-to-moderate Ru loading arises from the formation of surface oxide species on ultrasmall Ru nanoclusters, which exhibit a high surface-to-volume ratio and thus exhibit a greater fraction of exposed oxygen. As a result, a higher O/Ru ratio can be observed at the early stage of Ru deposition, particularly for Ru50, where Ru nanoclusters are optimally dispersed across the GMS framework. In contrast, excessive Ru deposition allows the growth and partial coalescence of Ru nanoclusters, as evidenced by SEM images (Figure 3), thereby increasing the relative contribution of metallic Ru and reducing the fraction of oxidized surface species. This effect led to a reduced O/Ru ratio for Ru100 and Ru150 samples. This evolution indicates a balance between Ru dispersion and surface chemistry, where an optimal Ru loading enables enhanced surface oxide formation without inducing Ru agglomeration or deterioration of the structurally robust GMS scaffold.

Figure 4c shows the high-resolution C 1s spectra, exhibiting the chemical states of carbon upon Ru depositions. The dominant peak at 284.8 eV is assigned to typical sp^2^ hybridized graphitic carbon, which confirms the graphene structure.^33^ Additional minor peaks located at 286.1, 288.9, and 290.4 eV are attributed to C–O, C=O, and O=C–O bonding environments, respectively.^33,34^ These features correspond to oxidized surface species that are introduced during Ru nucleation, as discussed previously.^35,36^ Importantly, the C 1s spectra of Ru/GMS samples exhibit no significant changes in peak positions and spectral components relative to those of pristine GMS, indicating that Ru deposition does not induce lattice disruption or oxidative damage to the graphene framework. This confirms the high structural robustness of the GMS scaffold and its ability to accommodate Ru nanoclusters, in which pre-existing topological defects function as stable and durable center sites for metal nucleation and deposition.^23,30^ This agrees well with the Raman analysis in Figure 4a. The presence of Ru species is further confirmed by Ru 3d signals, which are clearly observed for Ru50 and higher loading. Furthermore, a progressive shift of Ru 3d peaks toward lower binding energy (Δ*E* = ∼1.4 eV) is observed for Ru-rich samples (Ru150), which occurs from electronic interactions between Ru nanoclusters and the GMS support. This shift is attributed to charge redistribution at the Ru–C interface, which provides the electron-rich Ru centers for accelerating electron transfer kinetics during the HER.^9,10,36^ Such electronic modulation likely optimizes the *d*-band center for Ru species, giving balanced hydrogen adsorption and desorption, which are essential factors for enhancing the Volmer–Heyrovsky mechanism. Moreover, we also observed additional components associated with RuO_2_ (∼283 eV), suggesting partial surface oxidation during electrochemical deposition. The dual-phase configuration plays a crucial role in the HER, as metallic Ru facilitates hydrogen adsorption while oxide-formed Ru promotes water dissociation, thereby improving catalytic efficiency.^37,38^ These observations are consistent with previously reported carbon-supported Ru catalysts, such as Ru on carbon dots and carbon nanotubes.^9,10,36^

Additionally, the Ru 3p spectra of Ru/GMS samples with varying Ru loadings (Ru10–Ru150) are shown in Figure 4d. For Ru10, the Ru signal is barely detectable, referring to minimal Ru incorporation, which exceeds the detection limit of XPS analysis. At moderate loading (Ru50), two distinct peaks at 464.0 and 486.1 eV correspond to the 3p_3/2_ and 3p_1/2_ orbitals of metallic Ru^0^, while the two minor peaks at 469.7 and 489.1 eV are attributed to the 3p_3/2_ and 3p_1/2_ orbitals of Ru^3+^ species.^9,39^ It was clearly seen that the spectra are dominated by Ru^0^, indicating the presence of metallic Ru nanoclusters as the primary phase, which is consistent with the TEM images (Figure 2). With increasing Ru deposition (Ru150), a shift of Ru^0^ peaks toward lower binding energy becomes more pronounced (Δ*E* = ∼1.7 eV), confirming significant charge transfer from the graphene support to Ru, thereby enriching Ru with electrons (a trend consistent with the Ru 3d spectra shown in Figure 4c).^9,10,36^

### 3.3 Electrochemical Performance

To evaluate the hydrogen evolution activity, Figure 5a shows polarization curves of Ru/GMS electrodes with varied Ru loading (Ru2 to Ru150), benchmarked against pristine GMS and Pt in 0.5 M H_2_SO_4_. Pristine GMS performs negligible HER activity as it requires an overpotential of over −0.60 V vs RHE to reach 10 mA cm^–2^, indicative of poor intrinsic catalytic activity. In contrast, Ru incorporation (Ru/GMS) markedly enhances HER performances, as reflected by the substantial reduction in overpotentials. It was found that the overpotential at 10 mA cm^–2^ decreased to −0.25 V (Ru2), −0.19 V (Ru10), −0.13 V (Ru50), −0.13 V (Ru100), and −0.12 V (Ru150), as shown in Figure 5b. The trend demonstrates a sharp improvement up to Ru50, followed by a plateau beyond Ru100, suggesting no significant enhancement at excessive Ru loading. Note the HER measurements were conducted using at least three different electrodes to ensure reliability. To evaluate mechanistic insights into HER kinetics, Tafel slopes of Ru/GMS electrodes are shown in Figure 5c. It was found that Tafel values decreased from 254 mV dec^–1^ (GMS) to 82 mV dec^–1^ (Ru2), reaching 74 mV dec^–1^ for Ru10, and further to 64–67 mV dec^–1^ for Ru50–Ru150. This indicates that Ru incorporation significantly decreases Tafel slopes, which exhibits an accelerated HER pathway toward the Volmer–Heyrovsky mechanism.^12,15,40^ Among all samples, Ru50 exhibits the lowest Tafel slope, reflecting optimal Ru coverage that promotes rapid hydrogen adsorption and desorption processes. However, additional Ru depositions beyond Ru50 give negligible HER improvement, emphasizing again that Ru overloading limits the HER kinetics. These results underscore the critical role of controlled Ru loading to balance active site density and structural accessibility. Additionally, the mass activity-normalized polarization curves are shown in Figure 5d. At an overpotential of 150 mV, the mass activities of Ru/GMS increase markedly with increasing Ru loading, rising from 4 A g^–1^ (Ru2) to 300 A g^–1^ (Ru150), while commercial Pt/C delivers 800 A g^–1^. For a fair comparison with a carbon-supported Ru, Ru50 achieves a mass activity of ∼200 A g^–1^, nearly 3 times higher than that of Ru/Ketjen black (72 A g^–1^) at equivalent Ru deposition cycles (see Figure S6). This underscores the advantage of the GMS architecture; its hollow, sponge-like framework promotes efficient Ru utilization and enhances interfacial Ru-GMS charge transfer, leading to improving HER kinetics.

**Figure 5:**
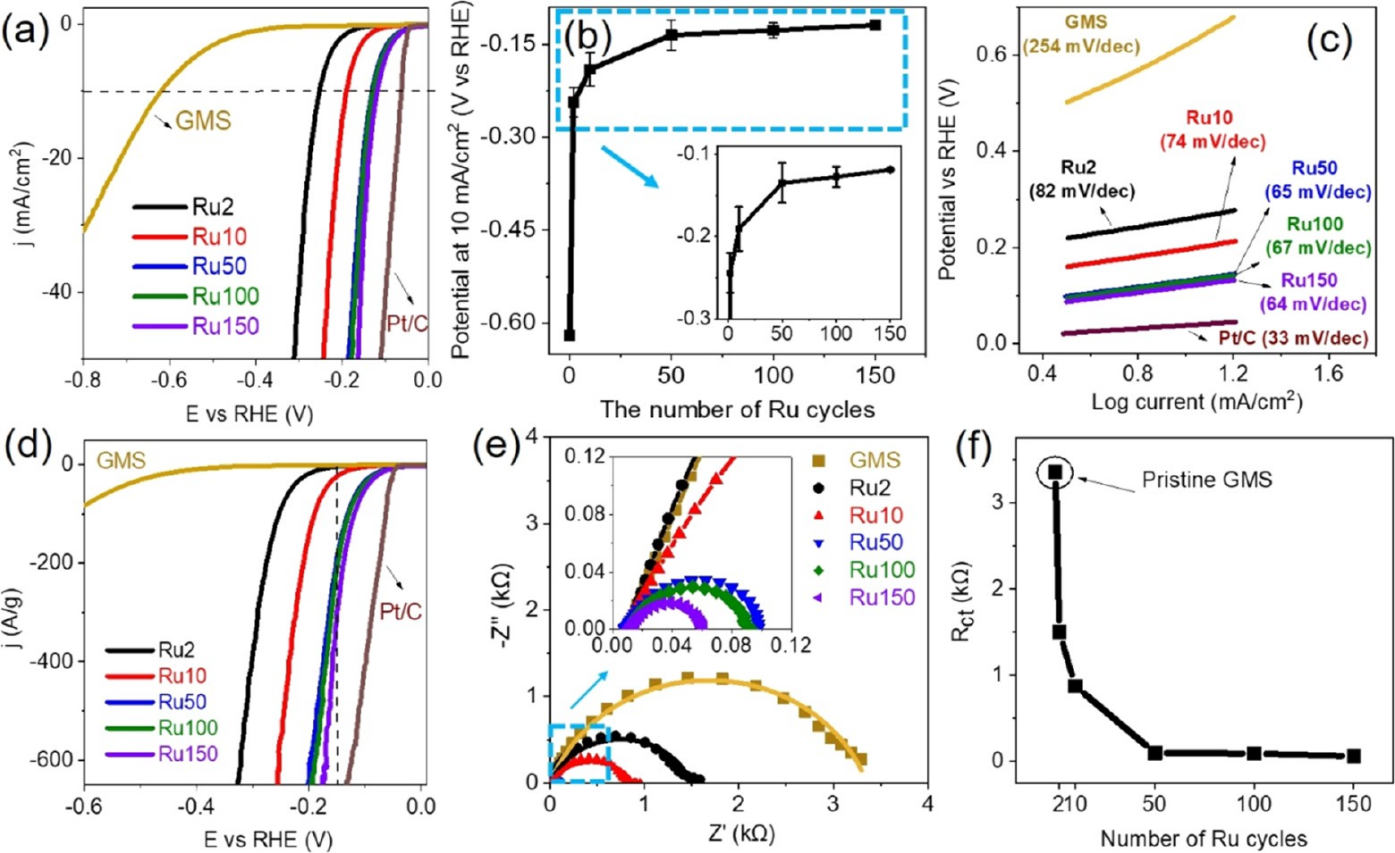
Electrochemical HER performance in 0.5 M H_2_SO_4_. (a) Polarization curves of Ru/GMS electrodes at varied Ru deposition cycles (Ru2–Ru150) compared to pristine GMS and Pt. (b) Overpotentials at 10 mA cm^–2^ as a function of Ru loading. (c) Tafel plots of Ru/GMS samples with their corresponding Tafel slopes. (d) Polarization curves of Ru/GMS normalized by mass activities. (e) Nyquist plots at a potential of −0.13 V vs RHE. (f) The decreasing trend of charge transfer resistance (*R*_ct_) as a function of Ru loadings. All electrodes were prepared on GCE with a constant mass loading of 100 μg cm^–2^.

Figure 5e shows Nyquist plots as obtained from electrochemical impedance spectroscopy (EIS) at a constant potential of −0.13 V vs RHE, providing characteristic charge transfer properties. The high-frequency intercept of the semicircle presents the solution resistance (*R*_s_), while the semicircle diameter corresponds to the charge transfer resistance (*R*_ct_). The Ru/GMS and pristine GMS samples exhibit similar *R*_s_ values of *ca*. 10 Ω, indicating comparable electrolyte resistance across electrodes, while *R*_ct_ decreases progressively with increasing Ru depositions. The *R*_ct_ values of the Ru/GMS electrodes are *ca*. ∼1490 Ω (Ru2), ∼870 Ω (Ru10), ∼90 Ω (Ru50), ∼80 Ω (Ru100), and ∼50 Ω (Ru150), compared to pristine GMS possessing a large semicircle. The trend as a function of Ru loadings is shown in Figure 5f. It is clearly seen that a steep decline in *R*_ct_ was observed up to Ru50, followed by a marginal improvement beyond this point. This suggests that excessive Ru coverage does not significantly enhance conductivity due to nanocluster aggregation, which can partially obstruct electrolyte diffusion. The superior HER performance of Ru50 (as evidenced by its low overpotential, minimal Tafel slope, and optimal *R*_ct_) is attributed to the synergistic balance between uniform Ru dispersion and strong interfacial coupling within the conductive GMS framework. This structural and electronic integration can potentially facilitate efficient electron transfer during HER, thereby accelerating reaction kinetics. These findings highlight the importance of rational Ru deposition on graphene-based porous materials to achieve excellent catalytic efficiency.

To evaluate the electrochemical capacitive properties, Figure 6a–d shows cyclic voltammetry (CV) profiles of pristine GMS and Ru-anchored GMS electrodes (Ru2–Ru150) recorded within the nonfaradaic potential range of 0.68–0.88 V vs RHE at various scan rates in 0.5 M H_2_SO_4_ electrolyte. Additional CV profiles of Ru10 and Ru100 are provided in Figure S7. It was found that all samples exhibit nearly rectangular CV curves, which demonstrate characteristic ideal capacitive behavior as current response increases proportionally with scan rates. Figure 6e compares CV profiles of all samples at a scan rate of 100 mV s^–1^, indicating a noticeable reduction in CV areas after Ru deposition. The linear plots of current density versus scan rates are shown in Figure 6f, where the slopes of the plots present areal capacitance. The areal capacitances were determined as 2.09 mF cm^–2^ (Ru2), 1.21 mF cm^–2^ (Ru10), 0.97 mF cm^–2^ (Ru50), 1.39 mF cm^–2^ (Ru100), and 1.52 mF cm^–2^ (Ru150), compared to pristine GMS possessing the highest capacitance of 2.15 mF cm^–2^ (see Figure 6f, inset). The superior capacitance of pristine GMS is attributed to its high surface area and abundant porosity. Interestingly, Ru incorporation significantly decreases capacitance up to Ru50, attributed to partial pore blockage by Ru nanoclusters that limit carbon exposure. This result agrees well with the surface analysis from the nitrogen adsorption–desorption isotherms in Figure 3h,i. In other words, the observed decrease in double-layer capacitance can be attributed to changes in the surface charge characteristics of the GMS framework induced by Ru deposition. Incorporation of Ru nanoclusters could modify the interfacial electronic environment, resulting in a shift of the Ru/GMS surface toward a less negatively charged state. This shift can reduce charge adsorption at the electrolyte–electrode interface, thereby leading to a lower measured capacitance. However, capacitance increases again at Ru100 and Ru150, reflecting the contribution of Ru nanoclusters that introduce additional electrochemically active surface area, leading to an increase in charge storage and interfacial activity. This trend demonstrates the critical interplay between Ru loading and accessible surface area, which significantly impacts double-layer capacitance and HER performance. While low Ru loading reduces capacitance due to pore obstruction, optimized and higher Ru loading can compensate by providing a metallic Ru surface that contributes to both capacitive behavior and catalytic activity.

**Figure 6:**
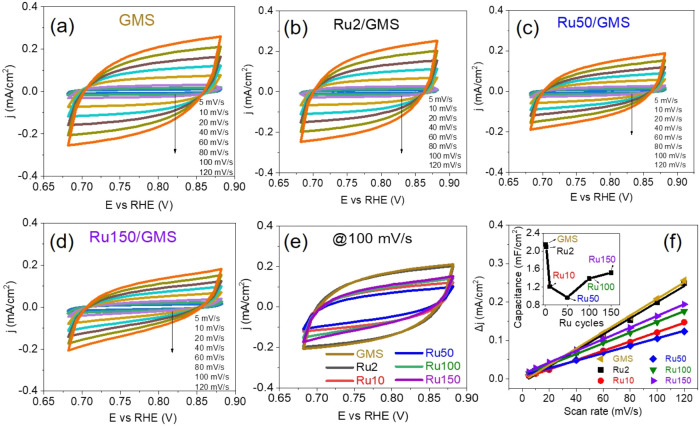
Electrochemical capacitive properties. (a–d) Cyclic voltammetry (CV) profiles of pristine GMS and various Ru samples recorded within the nonfaradaic potential range (0.68–0.88 V vs RHE) at various scan rates in 0.5 M H_2_SO_4_. (e) Comparing CV curves of all samples at a scan rate of 100 mV s^–1^. (f) Linear plots of the capacitive current density (Δ*J*/2) versus scan rate, where Δ*J* is the different current density between anodic and cathodic current densities. The inset in (f) shows the areal capacitances as a function of Ru deposition cycles, obtained from the slope of the linear plots.

To evaluate catalytic activity under practical conditions, the HER performance of Ru50 and pristine GMS was tested in 1 M KOH electrolyte, as shown in Figure 7a–c. It was found that Ru50 exhibits outstanding HER activity by achieving 10 mA cm^–2^ at an overpotential of only −0.061 V, whereas pristine GMS requires an overpotential exceeding −0.60 V (see Figure 7a). This activity approaches that of the Pt electrode that requires an overpotential of −0.052 V to reach 10 mA cm^–2^. Tafel analysis is shown in Figure 7b. The Tafel slope of Ru50 (88 mV dec^–1^) is substantially lower than that of pristine GMS (223 mV dec^–1^), which is close to the Pt activity (77 mV dec^–1^). This indicates that Ru50 facilitates the HER via the Volmer–Heyrovsky mechanism under an alkaline condition.^9,10,37^ In Figure 7c, capacitive analysis also reveals that the areal capacitance of Ru50 (1.3 mF cm^–2^) is slightly higher than that of pristine GMS (1.1 mF cm^–2^), supported by its larger CV profile (see Figure 7c, inset). This behavior may arise from the slightly less negatively charged state of Ru50, which can modestly enhance hydroxide adsorption and consequently lead to a marginal increase in capacitive characteristics. To assess catalyst durability and performance in a real-world environment, HER activity was further evaluated in neutral seawater, as shown in Figure 7d–f. It is clearly seen that Ru50 achieves a current of 10 mA cm^–2^ at an overpotential of *ca*. −0.39 V, while pristine GMS requires −1.12 V, indicating a dramatic enhancement under saline conditions (Figure 7d). Tafel slopes shown in Figure 7e confirm improved kinetics, with the Tafel value of Ru50 (256 mV dec^–1^) being substantially lower than that of pristine GMS (316 mV dec^–1^). This enhancement is attributed to the increase in areal capacitance of Ru50 (0.9 mF cm^–2^), which is nearly eight times higher than that of pristine GMS (0.12 mF cm^–2^), see linear plots and CV profiles in Figure 7f. It should be noted that the *C*_dl_ of pristine GMS in seawater is substantially lower than in acidic (2.15 mF cm^–2^) and alkaline (1.10 mF cm^–2^) media (Table S1), consistent with the well-studied inverse correlation between the *C*_dl_ and the hydrated size of ions.^41^ Interestingly, the ion-size effect could be less pronounced for Ru50. This is because the *C*_dl_ of Ru50 in seawater (0.9 mF cm^–2^) remains comparable to its capacitance in acidic (0.97 mF cm^–2^) and alkaline (1.3 mF cm^–2^) electrolytes. This behavior suggests that Ru nanoclusters anchored on the GMS scaffold promote more favorable interfacial interactions with diverse ion species regardless of their hydrated radii. The presence of Ru nanoclusters likely modifies the local surface charge and provides additional accessible active sites, leading to the stabilization of interfacial capacitance even in the ion-rich environment of seawater. Therefore, these results highlight the critical role of Ru nanoclusters in mitigating corrosion and maintaining HER activity, positioning Ru50-modified GMS as a highly promising candidate for seawater electrolysis. Additional CV profiles of Ru50 and pristine GMS at various scan rates in alkaline and seawater electrolytes are presented in Figure S8. To further assess HER performance, the mass activity-normalized polarization curves in alkaline and seawater electrolytes are shown in Figure S9. In alkaline media, Ru50 achieves a mass activity of 320 A g^–1^, closely approaching that of Pt/C (380 A g^–1^) at an overpotential of 150 mV. In seawater, Ru50 similarly demonstrates competitive activity, delivering 113 A g^–1^ compared with 138 A g^–1^ for Pt/C at 400 mV. These results highlight the strong applicability of Ru/GMS for efficient HER across diverse environments.

**Figure 7:**
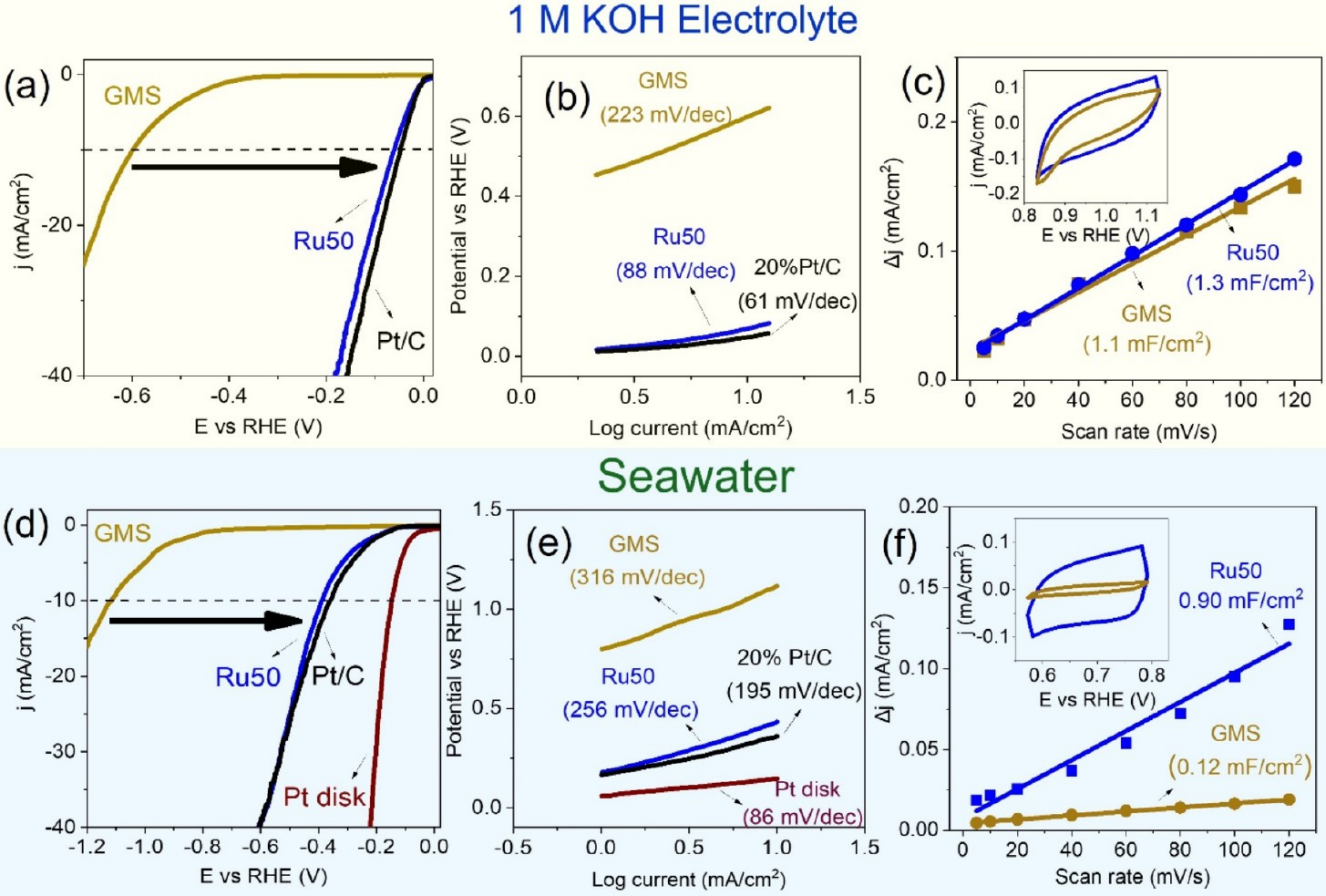
HER performance of Ru50/GMS in alkaline and seawater electrolytes. Polarization curves of Ru50 and pristine GMS with corresponding Tafel slopes in (a, b) 1 M KOH and (d, e) neutral seawater. Linear plots of the capacitive current density versus scan rates (5–120 mV s^–1^) with their areal capacitance in (c) 1 M KOH and (f) seawater; insets compare CV curves of Ru50 and the GMS at a scan rate of 100 mV s^–1^.

To provide a fair comparison, we also evaluated the HER performance of Ru deposited on commercial Ketjen black (EC-300J) at an equivalent Ru loading. Ru/GMS exhibits markedly superior activity relative to Ru/Ketjen black in acidic and alkaline media and comparable performance in seawater (Figure S6). These findings underscore the advantages of the GMS architecture, in which a hollow, sponge-like framework promotes efficient Ru utilization and enhances interfacial Ru-GMS electronic coupling. Comparison of the HER performance of the Ru/GMS catalysts with other Ru-containing carbon electrocatalysts is shown in Table S2.

To comprehensively evaluate the long-term stability of Ru/GMS catalysts under prolonged electrochemical operation, Figure 8 shows HER performance before and after the stability test, along with postcharacterizations. Figure 8a compares the polarization curves of Ru50 before and after 5000 CV cycles in a potential range of 0.68–0.88 V vs RHE at a scan rate of 100 mV s^–1^. The comparative analysis of these curves provides insight into electrochemical durability, particularly the stability of the charge/discharge behavior of the catalyst surface and the preservation of ion-accessible active surface sites.^12,15^ Remarkably, the curves exhibit negligible deviation, indicating that Ru50 maintains catalytic activity even after extensive cycling. Moreover, catalysts’ durability under ultrahigh current density was examined using chronoamperometry (Figure 8b), where Ru50 and Ru100 were operated at a constant potential of −0.35 V vs RHE for 24 h. It was found that Ru50 exhibited superior stability, giving sustainability for a current density of −208 ± 10 mA cm^–2^ with minimal fluctuation, whereas Ru100 provided comparatively lower and less stable HER performance with a fluctuating current density of −151 ± 27 mA cm^–2^. The excellent durability of Ru50 is attributed to its optimized Ru dispersion and strong interfacial Ru bonding with the GMS support, leading to minimal agglomeration and corrosion under high current conditions. In contrast, Ru100 possessing excessive Ru loading may promote partial detachment and agglomeration of Ru nanoclusters, resulting in a reduction in long-term stability.^11,12^ To observe the physical properties of catalysts after testing, poststability characterizations, including PXRD, SEM, and EDX analyses, are shown in Figure 8c–g. Figure 8c compares the PXRD patterns of Ru50 before and after testing. Both patterns exhibit a characteristic peak of the (002) plane of graphitic carbon at 2θ of 26.6°, with no observable changes in peak position and shape. This confirms that the GMS framework, as a catalyst support, retains its crystalline integrity even after prolonged electrochemical operation. The absence of additional diffraction peaks (e.g., graphene oxide or RuO_2_ (JCPDS 40–1290)) with structural distortion strongly highlights the robustness of Ru anchoring and the resilience of the GMS scaffold during prolonged operation. Moreover, morphologies and elemental mapping of Ru50 after testing are shown in Figure 8d–g. The SEM image in Figure 8d shows that the sponge-like GMS structure is retained, with no evidence of electrode collapse. The EDX mappings of C, O, and Ru in Figure 8e–g confirm the presence of Ru across the GMS framework, with localized regions of higher Ru intensity that are consistent with slight Ru aggregation after the stability test. Moreover, electrode durability after long-term exposure in diverse electrolytes was also investigated, as discussed in Figure S10. These results indicate that Ru50 exhibits outstanding structural and electrochemical stability, demonstrating the effectiveness of surface-engineered Ru anchoring in preventing detachment or structural degradation during extended HER operations.

**Figure 8:**
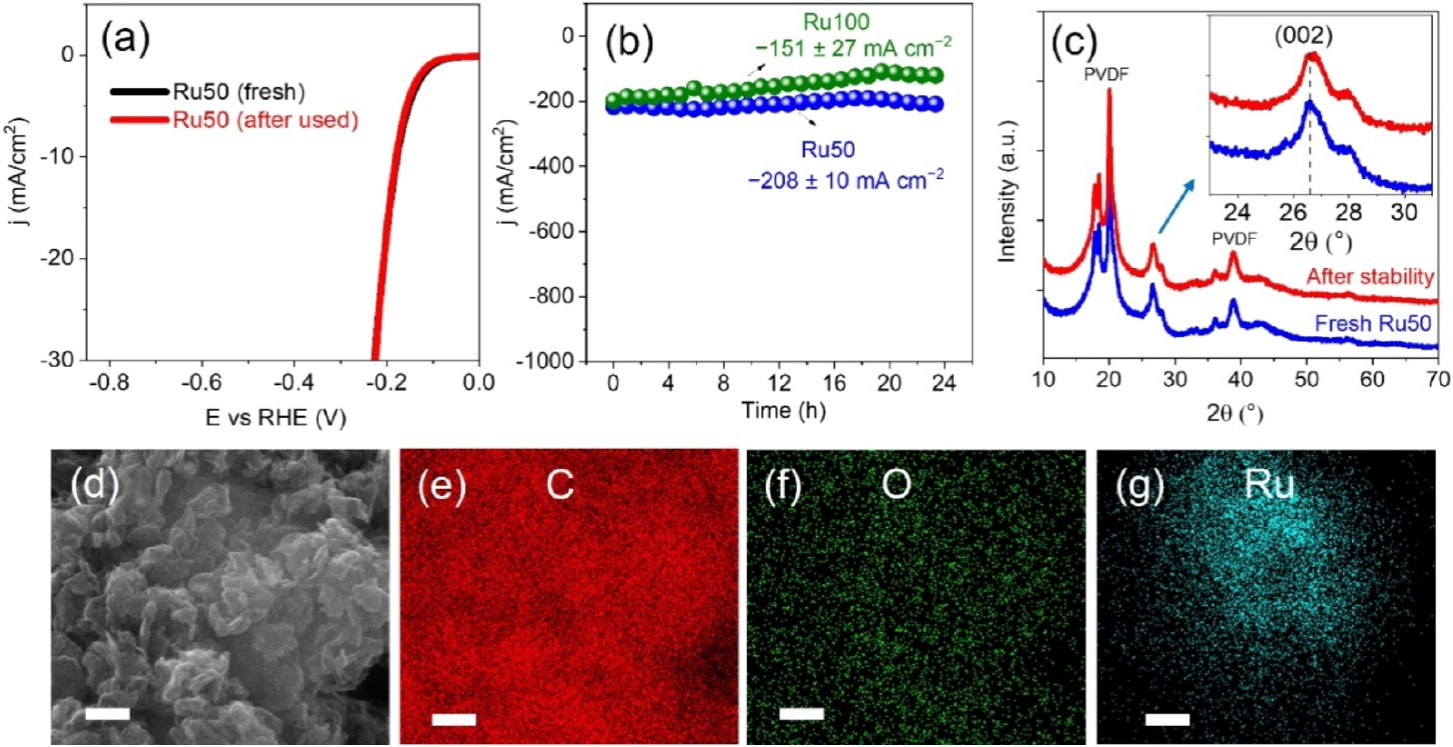
Stability test of Ru/GMS catalysts. (a) Polarization curves before and after 5000 CV cycles (0.68–0.88 V vs RHE) at a scan rate of 100 mV s^–1^. (b) Comparing chronoamperometry of Ru50 and Ru100 at −0.35 V vs RHE for 24 h. (c) PXRD pattern of Ru50 before and after stability testing. Inset in (c) compares the (002) diffraction pattern of GMS, showing no change in crystalline structure. (d) SEM images of Ru50 after stability testing with EDX elemental mapping showing (e) C, (f) O, and (g) Ru. All scale bars are 1 μm.

## 4 Conclusions

In conclusion, we have demonstrated the successful design of a surface-engineered graphene mesosponge (GMS) decorated with uniformly dispersed Ru nanoclusters as an advanced electrocatalyst for the hydrogen evolution reaction (HER). Comprehensive structural and electronic analyses reveal that the hierarchical porosity of GMS, coupled with strong Ru–graphene interfacial bonding and electronic modulation, synergistically enhances the catalytic activity. The optimized Ru50/GMS exhibits exceptional HER performance across acidic, alkaline, and seawater electrolytes, achieving low overpotentials and accelerated kinetics while maintaining remarkable catalyst stability under prolonged cycling and ultrahigh current densities. Therefore, these findings underscore the pivotal role of rational Ru loading and graphene-based porous frameworks in enabling pH-universal hydrogen production. Therefore, this study provides a scalable strategy for efficient and sustainable green hydrogen production, offering significant implications for the advancement of future energy conversion technologies.

## Supplementary Material



## Notes

Further sample characterizations, including HR-TEM, SEM, XRF, XPS, and further electrochemical analysis with comparing HER performance (PDF)
