# Supplementary material for: Surface-Engineered Ru–Graphene Mesosponge Catalysts for pH-Universal and Seawater Hydrogen Evolution
Source: ACS Nanosci Au. 2026 Mar 28;6(3):491–504. doi: 10.1021/acsnanoscienceau.6c00016 (PMC13281174; doi:10.1021/acsnanoscienceau.6c00016)
Supplement: Supplementary file 1 [file ng6c00016_si_001.pdf]

Supporting Information

Surface-Engineered Ru–Graphene Mesosponge Catalysts for pH-Universal and Seawater Hydrogen Evolution

Nichakarn Sornnoei [a], Naruewan Samantarkun [a], Thanit Saisopa [b], Panwad Chavalekvirat [c], Pawin Iamprasertkun [c], Bin Wang [d], Tawan Sooknoi [e], Shinichiroh Iwamura [f, g], Hirotomo Nishihara [g*], Wisit Hirunpinyopas [a*]

[a] *Department of Chemistry and Center of Excellence for Innovation in Chemistry, Faculty of Science, Kasetsart University, Chatuchak, Bangkok, 10900, Thailand*

[b] *Department of Applied Physics, Faculty of Sciences and Liberal Arts, Rajamangala University of Technology Isan, Nakhon Ratchasima, 30000, Thailand*

[c] *School of Bio-Chemical Engineering and Technology, Sirindhron International Institute of Technology, and Research Unit in Sustainable Electrochemical Intelligent, Thammasat University, Pathum Thani 12120, Thailand*

[d] *State Key Laboratory of Heavy Oil Processing, College of Chemistry and Chemical Engineering, China University of Petroleum (East China), Qingdao, 266580, China*

[e] *Department of Chemistry, School of Science, King Mongkut's Institute of Technology Ladkrabang, Chalongkrung Road, Ladkrabang, Bangkok 10520, Thailand*

[f] *Faculty of Symbiotic Systems Science, and Hydrogen Energy Research Institute (HERI), Fukushima University, Fukushima 9601296, Japan*

[g] *Advanced Institute for Materials Research (WPI-AIMR), Tohoku University, Sendai 9808577, Japan*

*Corresponding authors:

Hirotomo Nishihara (hirotomo.nishihara.b1@tohoku.ac.jp)

Wisit Hirunpinyopas (wisit.hi@ku.ac.th)

Contents

S1. LSV of Ru depositions

S2. TEM images and elemental mapping of Ru/GMS

S3. XRF spectrum of Ru/GMS

S4. SEM images and elemental mapping of Ru/GMS

S5. XPS analysis

S6. Comparing HER performance between Ru/GMS and Ru/Ketjen black

S7. Further CV curves of Ru/GMS in various electrolytes

S8. HER activity based on mass activities

S9. Electrode durability after long-term exposure

S10. Supporting references

S1. LSV of Ru depositions

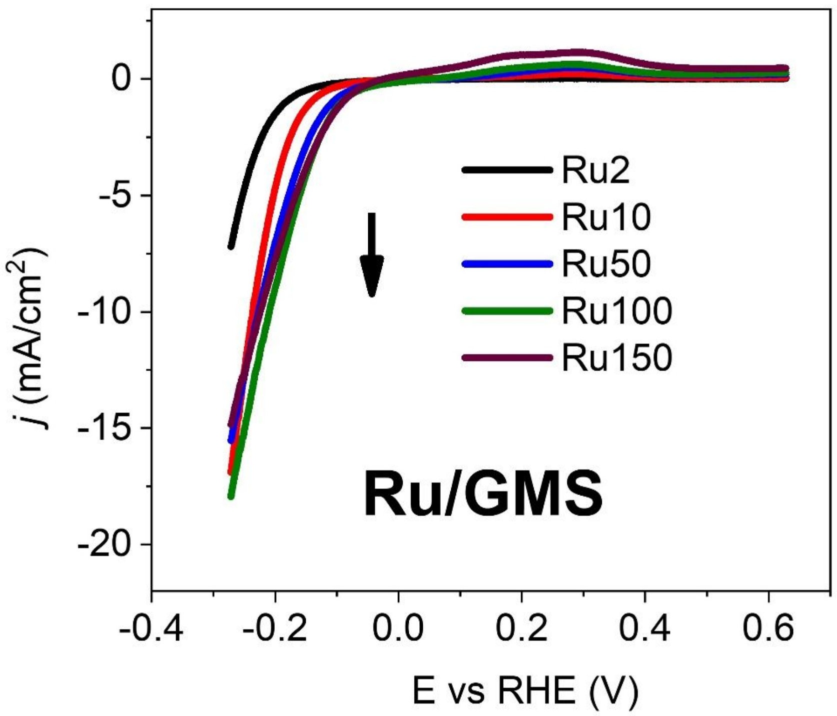

Figure S1. LSV showing the number of Ru deposition cycles on GMS supports from Ru2 (2 Ru deposition cycles) to Ru150 (150 Ru deposition cycles).

S2. TEM images and elemental mapping of Ru/GMS

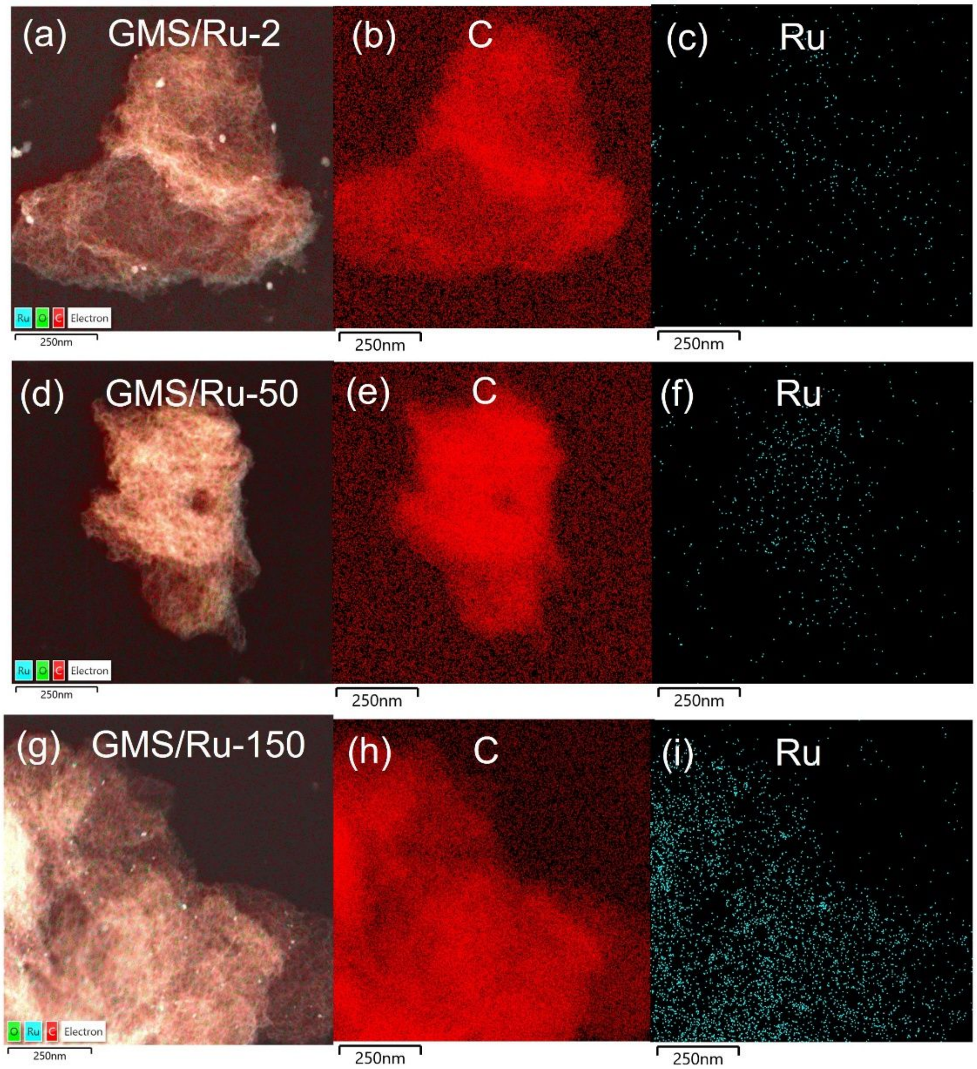

Figure S2. TEM images and elemental mapping of (a-c) Ru2/GMS, (d-f) Ru50/GMS, (g-i) Ru150/GMS, showing the well dispersed Ru decoration on GMS support.

S3. XRF spectrum of Ru/GMS

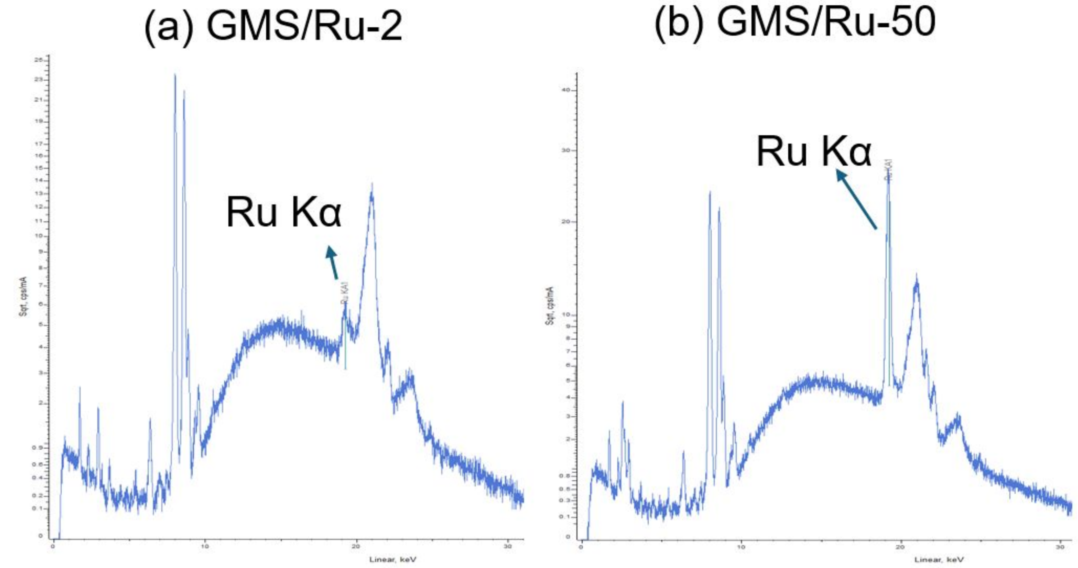

Figure S3. XRF spectrum of (a) Ru2/GMS and Ru50/GMS, showing the Ru peak.

S4. SEM images and elemental mapping of Ru/GMS

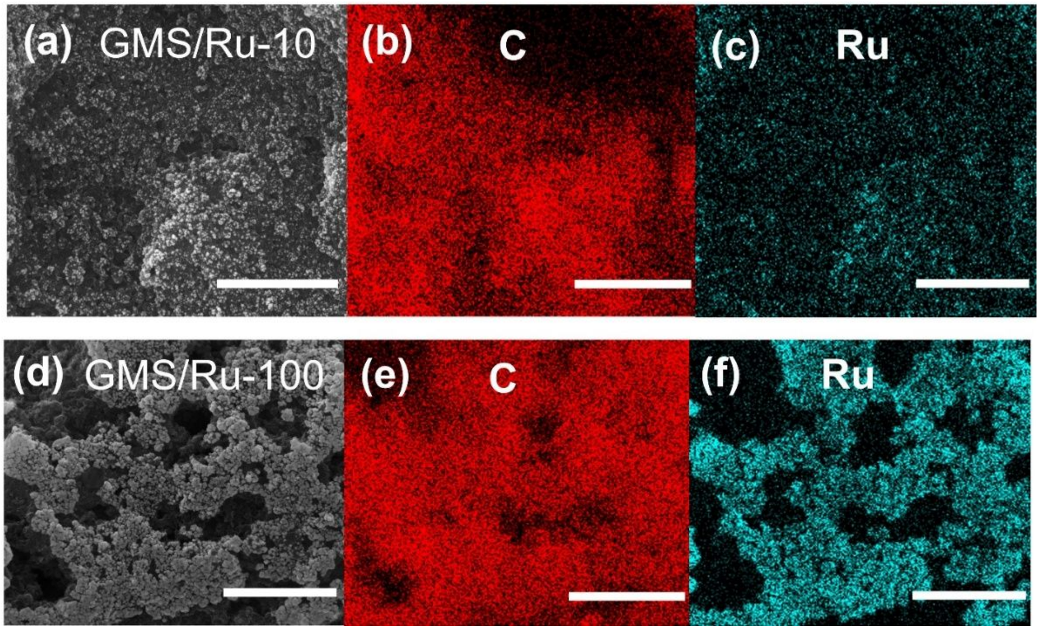

Figure S4. SEM images and elemental mapping of (a-c) Ru2/GMS, (d-f) Ru50/GMS, (g-i) Ru150/GMS, showing the well dispersed Ru decoration on GMS support. Note all scale bars are 10 μm.

S5. XPS analysis

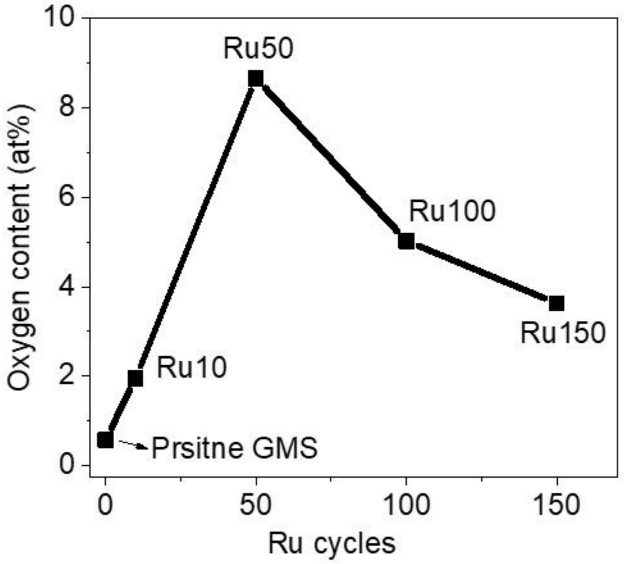

Figure S5. Oxygen content of Ru/GMS at varying Ru deposition cycles as determined from XPS analysis.

S6. Comparing HER performance between Ru/GMS and Ru/Ketjen black

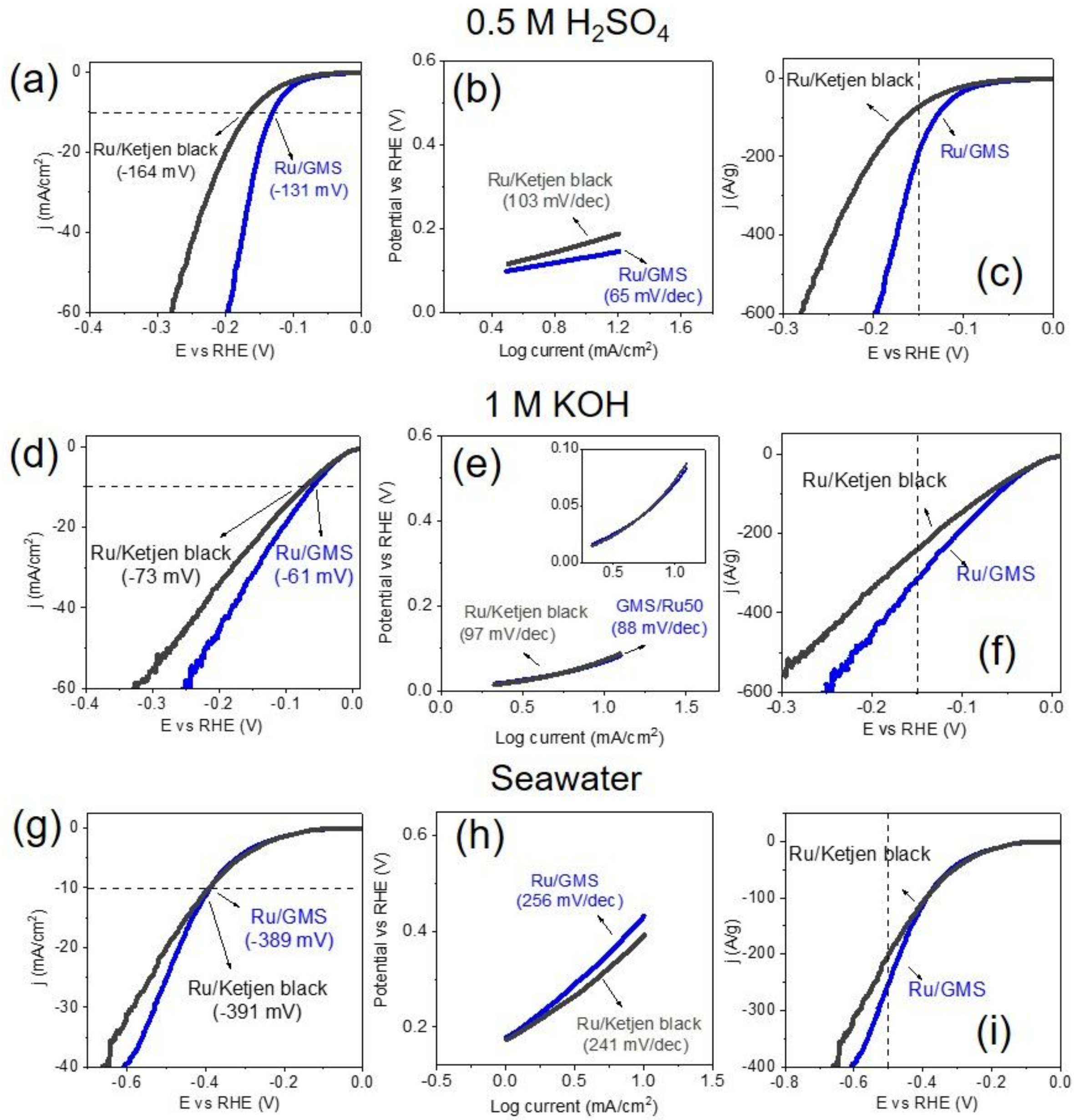

Figure S6. HER comparison between Ru/GMS with Ru/Ketjen black at equivalent Ru loading (50 deposition cycles). Polarization curves normalized by (a, d, g) area of gassy carbon electrode and normalized by (c, f, i) mass activities in 0.5 M H_2SO_4, 1 M KOH, and seawater; and (b, e, h) their corresponding Tafel plots.

S7. Further CV curves of Ru/GMS in various electrolytes

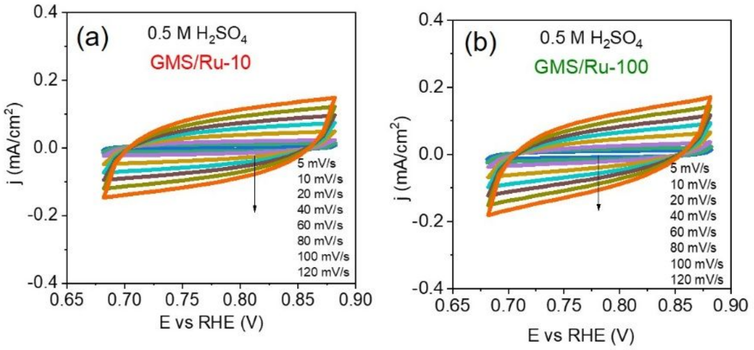

Figure S7. Cyclic voltammetry (CV) profiles of (a) Ru10 and (b) Ru100 recorded within the nonfaradaic potential range (0.68-0.88 V vs. RHE) at various scan rates in 0.5 M H_2SO_4 electrolyte.

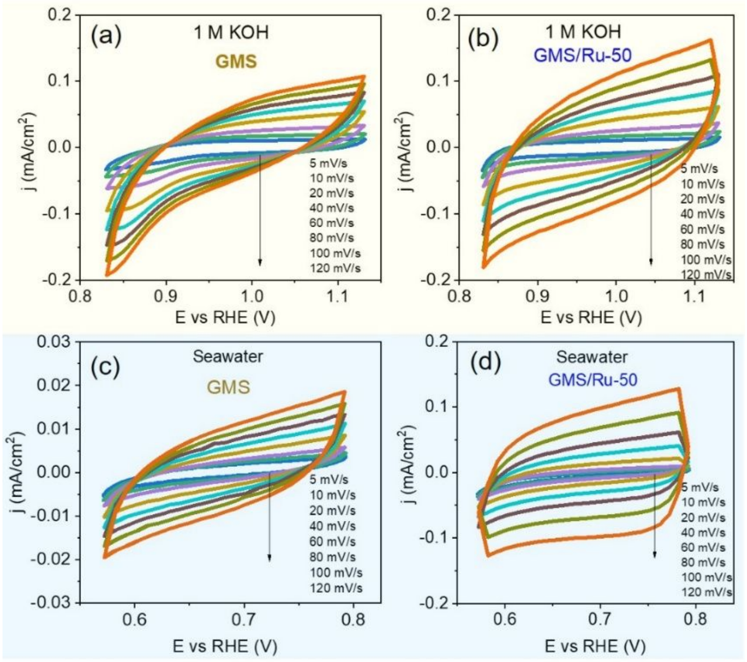

Figure S8. CV profiles at various scan rates of (a) pristine GMS and (b) Ru50 recorded within 0.8-1.1 V vs. RHE in 1 M KOH electrolyte. CV profiles at various scan rates of (c) pristine GMS and (d) Ru50 recorded within 0.6-0.8 V vs. RHE in neutral seawater electrolyte.

Table S1. Double-layer capacitance (C_{dl}) of Ru/GMS samples in various electrolytes.

Catalyst	C_{dl} in 0.5 M H_2SO_4 (mF/cm^2)	C_{dl} in 1 M KOH (mF/cm^2)	C_{dl} in Seawater (mF/cm^2)
**Pristine GMS**	**2.15**	**1.10**	**0.12**
Ru2	2.09	-	-
Ru10	1.21	-	-
**Ru50**	**0.97**	**1.30**	**0.90**
Ru100	1.39	-	-
Ru150	1.52	-	-

Table S2. Comparison of the catalytic performance of the Ru/GMS catalysts with other Ru-containing carbon electrocatalysts for HER in diverse electrolytes.

Catalyst	0.5 M H_2SO_4		1 M KOH		Seawater		Ref.
	η_{10} (mV)	Tafel slope (mV/dec)	η_{10} (mV)	Tafel slope (mV/dec)	η_{10} (mV)	Tafel slope (mV/dec)	
[a] Ru/graphene	−220	77	>−200	148	/	/	[1]
[b] Ru@RuO_2/CNT	−272	116	/	/	/	/	[2]
[c] Ru/C (vulcan)	/	/	−102	60	/	/	[3]
[d] Ru/C	/	/	−112	~70	/	/	[4]
[e] c-CNT-0.68@TpBpy-Ru	/	/	−112	160	/	/	[5]
[f] Ru-MoS_2/CNT	/	/	−50	62	/	/	[6]
[g] Ru_1CoP/CDs	/	/	−51	73	/	/	[7]
[h] Ru@CQDs<br>[i] Ru@P-AC<br>5 wt% Ru/C	/	/	−65<br>−127<br>−94	63<br>103<br>66	/	/	[8]
Ru-CNTs<br>[j] RuCu-CNTs	/	/	−131<br>−39	136<br>89	/	/	[9]
[k] $Ru_{2.0}$/HNCS	−39	30	−72	67	/	/	[10]
[l] 3.0 wt%Ru/rGO	/	/	−112	31	/	/	[11]
[m] $RuYO_{2-x}$/C	/	/	−56	63	/	/	[12]
**Pristine GMS**	**−621**	**254**	**−600**	**223**	**−1116**	**316**	**This work**
**Ru2/GMS**	**−253**	**82**	/	/	/	/	**This work**
**Ru10/GMS**	**−191**	**74**	/	/	/	/	**This work**
**Ru50/GMS**	**−131**	**65**	**−61**	**88**	**−389**	**256**	**This work**
**Ru100/GMS**	**−129**	**67**	/	/	/	/	**This work**
**Ru150/GMS**	**−120**	**64**	/	/	/	/	**This work**
**20%Pt/C**	**−63**	**33**	**−48**	**61**	**−360**	**195**	**This work**
**Ru50/Ketjen black (EC-300J)**	**−164**	**103**	**−73**	**97**	**−391**	**241**	**This work**

Note [a] graphene was prepared via electrochemical cathodic exfoliation, [b] Ru@RuO_2 on carbon nanotubes, [c] 10 wt % Ru on carbon black (Vulcan XC72R), [d] Pure Ru supported on carbon, [e] Ru incorporated in CNT with bipyridine-based COF (TpBpy), [f] Ru-doped MoS_2 nanosheets closely sheathing around CNT, [g] CoP nanoparticles doped with Ru single-atom sites supported on carbon dots (CDs), [h] Ru nanoparticles supported on Porphyra-CQDs, [i] Ru nanoparticles on Porphyra activated carbon, [j] CuRu alloys on CNTs via flash Joule heating, [k] Ru nanoparticles within hollow nitrogen-doped carbon spheres, [l] Ru nanoparticles on reduced graphene oxide, [m] bimetallic $RuYO_{2-x}$ nanoparticles on graphene. η_{10} is a potential (mV) to acquire 10 mA cm^{-2}.

S8. HER activity based on mass activities

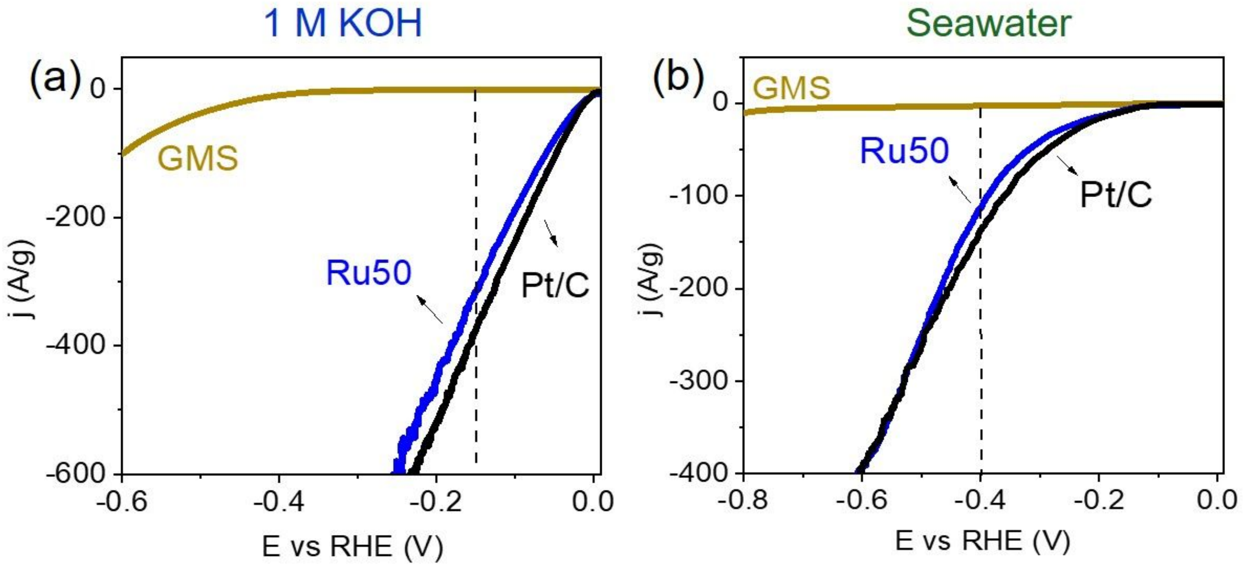

Figure S9. Comparing polarization curves normalized by mass activities of Ru/GMS and Pt/C in (a) 1 M KOH and (b) seawater.

S9. Electrode durability after long-term exposure

To evaluate catalyst durability, the Ru50 sample supported on a PVDF filter was immersed in DI water, 0.5 M H_2SO_4, 1 M KOH, and neutral seawater for 5 days (Figure S10a-b). After prolonged exposure, the Ru/GMS film remained fully intact with no observable detachment from the substrate, indicating excellent structural stability under harsh environments. Subsequent characterizations of the post-immersion samples using PXRD, SEM, EDX, and ICP-OES further confirmed this robustness. The PXRD patterns show unchanged (002) diffraction peak of GMS at 2θ of 26.6° (Figure S10c), with no detectable RuO_2-related peaks (JCPDS: 40-1290), demonstrating the absence of oxidation or phase transformation. EDX elemental mapping also reveals dispersed Ru species across the electrode surface, indicating strong anchoring Ru nanoclusters on GMS surface (Figure S10d-f). Moreover, ICP-OES analysis of the post-immersion electrolytes detected no Ru species, confirming negligible Ru leaching from the GMS scaffold.

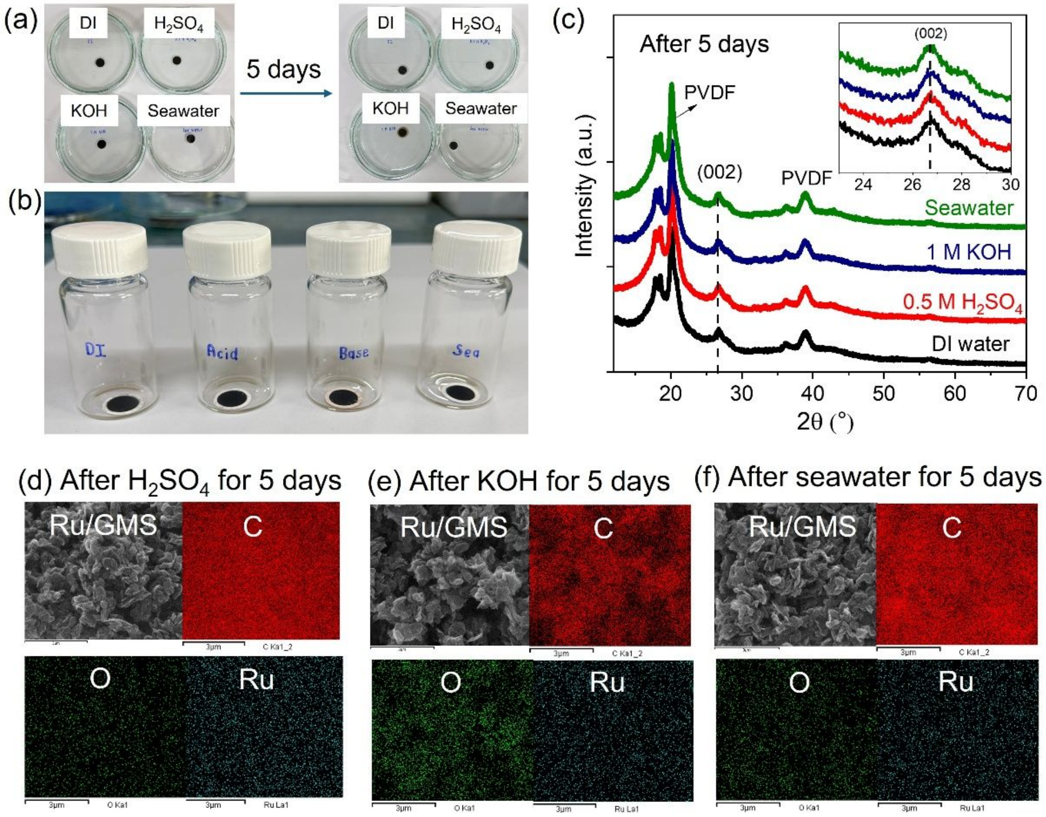

Figure S10. Stability test of the Ru/GMS samples. (a) Photographs of Ru/GMS supported on a PVDF filter after immersion in various electrolytes; DI water, 0.5 M H_2SO_4, 1 M KOH, and neutral seawater for 5 days. (b) Corresponding post-immersion appearance of the Ru/GMS samples. (c) Comparison of PXRD patterns of Ru/GMS after 5-day exposure to each electrolyte. SEM images and EDX elemental mapping of Ru/GMS following stability tests in (d) H_2SO_4, (e) KOH, and (f) seawater.

S10. Supporting references

(1) Qiang, L.; Bai, M.; Liu, Z.; Zhao, P.; He, S.; Zhao, M.; Yan, Q.; Wen, W.; Guo, Q.; Zhang, Y.; et al. Carbon Dot-Driven Spatial and Electronic Modulation of Ru on Graphene for pH-Universal Hydrogen Evolution Reaction Electrocatalysts. *Green Chem.* **2025**, *27* (39), 12364-12377. DOI: 10.1039/D5GC03796D.
(2) Romero, N.; Fenoll, D. A.; Gil, L.; Campos, S.; Creus, J.; Martí, G.; Heras-Domingo, J.; Collière, V.; Mesa, C. A.; Giménez, S.; et al. Ru-Based Nanoparticles Supported on Carbon Nanotubes for Electrocatalytic Hydrogen Evolution: Structural and Electronic Effects. *Inorg. Chem. Front.* **2023**, *10* (20), 5885-5896. DOI: 10.1039/D3QI00698K.
(3) Smiljanić, M.; Bele, M.; Pavko, L.; Hrnjić, A.; Ruiz-Zepeda, F.; Bijelić, L.; Kamšek, A. R.; Nuhanović, M.; Marsel, A.; Gašparič, L.; et al. Titanium Oxynitride-Supported Ru Nanoparticles as Exceptional Electrocatalysts for Alkaline Hydrogen Evolution Reaction. *Chem. Eng. J.* **2025**, *517*, 164204. DOI: 10.1016/j.cej.2025.164204.
(4) Zheng, Y.; Jiao, Y.; Zhu, Y.; Li, L. H.; Han, Y.; Chen, Y.; Jaroniec, M.; Qiao, S.-Z. High Electrocatalytic Hydrogen Evolution Activity of an Anomalous Ruthenium Catalyst. *J. Am. Chem. Soc.* **2016**, *138* (49), 16174-16181. DOI: 10.1021/jacs.6b11291.
(5) Sun, X.; Hu, Y.; Fu, Y.; Yang, J.; Song, D.; Li, B.; Xu, W.; Wang, N. Single Ru Sites on Covalent Organic Framework-Coated Carbon Nanotubes for Highly Efficient Electrocatalytic Hydrogen Evolution. *Small* **2024**, *20* (3), 2305978. DOI: 10.1002/smll.202305978.
(6) Zhang, X.; Zhou, F.; Zhang, S.; Liang, Y.; Wang, R. Engineering MoS_2 Basal Planes for Hydrogen Evolution via Synergistic Ruthenium Doping and Nanocarbon Hybridization. *Adv. Sci.* **2019**, *6* (10), 1900090. DOI: 10.1002/advs.201900090.
(7) Song, H.; Wu, M.; Tang, Z.; Tse, J. S.; Yang, B.; Lu, S. Single Atom Ruthenium-Doped CoP/CDs Nanosheets via Splicing of Carbon-Dots for Robust Hydrogen Production. *Angew. Chem. Int. Ed.* **2021**, *60* (13), 7234-7244. DOI: 10.1002/anie.202017102.
(8) Li, W.; Wei, Z.; Wang, B.; Liu, Y.; Song, H.; Tang, Z.; Yang, B.; Lu, S. Carbon Quantum Dots Enhanced the Activity for the Hydrogen Evolution Reaction in Ruthenium-Based Electrocatalysts. *Materials Chemistry Frontiers* **2020**, *4* (1), 277-284, 10.1039/C9QM00618D. DOI: 10.1039/C9QM00618D.
(9) Wei, W.; Li, P.; Lu, F.; Fan, K.; Li, B.; Wei, Y.; Zong, L.; Wang, L. Loading Uniformly Distributed CuRu Alloys on Carbon Nanotubes via Flash Joule Heating for High-Performance Electrocatalytic Water Splitting. *J. Alloys Compd.* **2023**, *936*, 168349. DOI: 10.1016/j.jallcom.2022.168349.
(10) Jiang, A.; Wang, Z.; Li, Q.; Dong, M. An Efficient Ruthenium-Based Dual-Electrocatalyst Towards Hydrogen Evolution and Oxygen Reduction Reactions. *Materials Today Physics* **2021**, *16*, 100300. DOI: 10.1016/j.mtphys.2020.100300.
(11) Feng, Y.; Zhang, S.; Zhu, L.; Li, G.; Zhao, N.; Zhang, H.; Chen, B. H. Reduced Graphene Oxide-Supported Ruthenium Nanocatalysts for Highly Efficient Electrocatalytic Hydrogen Evolution Reaction. *Int. J. Hydrogen Energy* **2022**, *47* (94), 39853-39863. DOI: 10.1016/j.ijhydene.2022.09.154.
(12) Li, X.; Deng, W.; Weng, Y.; Zhang, J.; Mao, H.; Lu, T.; Zhang, W.; Yang, R.; Jiang, F. Implanting H_xYO_{2-x} Sites into Ru-Doped Graphene and Oxygen Vacancies for Low-Overpotential Alkaline Hydrogen Evolution. *NPG Asia Mater.* **2023**, *15* (1), 55. DOI: 10.1038/s41427-023-00501-z.
